# Observation of the hyperfine structure and anticrossings of hyperfine levels in the luminescence spectra of LiYF_4_:Ho^3+^

**DOI:** 10.1038/s41377-022-00933-2

**Published:** 2022-08-02

**Authors:** Kirill N. Boldyrev, Boris Z. Malkin, Marina N. Popova

**Affiliations:** 1 Institute of Spectroscopy, Russian Academy of Sciences, Troitsk, Moscow, 108840 Russia; 2 Kazan Federal University, Kazan, 420008 Russia

**Keywords:** Fluorescence spectroscopy, Optical materials and structures

## Abstract

Resolved hyperfine structure and narrow inhomogeneously broadened lines in the optical spectra of a rare-earth-doped crystal are favorable for the implementation of various sensors. Here, a well-resolved hyperfine structure in the photoluminescence spectra of LiYF_4_:Ho single crystals and the anticrossings of hyperfine levels in a magnetic field are demonstrated using a self-made setup based on a Bruker 125HR high-resolution Fourier spectrometer. This is the first observation of the resolved hyperfine structure and anticrossing hyperfine levels in the luminescence spectra of a crystal. The narrowest spectral linewidth is only 0.0022 cm^−1^. This fact together with a large value of the magnetic *g* factor of several crystal-field states creates prerequisites for developing magnetic field sensors, which can be in demand in modern quantum information technology devices operating at low temperatures. Very small random lattice strains characterizing the quality of a crystal can be detected using anticrossing points.

## Abstract (web summary)

The first observation of hyperfine and isotope structures, deformation splitting, and level anticrossing in luminescence spectra of crystals is reported, on the example of LiYF4:Ho. All-optical magnetic field sensor is proposed.

## Graphical abstract

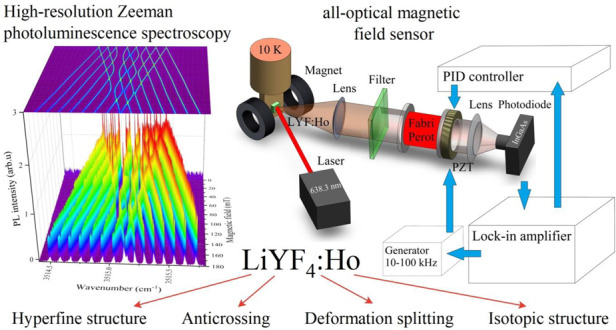

## Introduction

Crystals doped with rare-earth (RE) ions exhibit very narrow homogeneous and inhomogeneous linewidths of the 4f^N^–4f^N^ optical transitions, since the 4f^N^ electronic shell is well shielded from the crystalline environment by the filled 5s and 5p shells. The narrow-line spectra of transitions within the 4f^N^ shell of triply ionized RE elements cover the entire visible and infrared range. RE-doped materials are widely used as laser media, phosphors, scintillators, in solar cells, etc. Nowadays, RE-based luminescence thermometry is successfully developing, demonstrating a wide working temperature range, high thermal sensitivity, and spatial resolution^1,2^. Over the past decade, significant progress has been achieved in the application of RE-doped crystals for quantum information processing, which is based on the use of electronic-nuclear hyperfine levels^3–8^. Information on the hyperfine and superhyperfine interactions, isotopic effects, inhomogeneous line shapes, and random lattice strains in a crystal is essential for applications in modern quantum technologies. It can be acquired using high-resolution optical spectroscopy. High-resolution studies also reveal the narrowest spectral lines, which are favorable for sensor applications.

A series of high-resolution studies of RE materials has been performed using absorption or photoluminescence (PL) excitation spectroscopies. Hyperfine^7,9–25^, superhyperfine^24^, and isotope^23–30^ structures, as well as specific line shapes imposed by random lattice strains^23,31,32^ and the hyperfine level anticrossings in a magnetic field^33^ were observed in optical absorption spectra of a number of RE-doped crystals. We are not aware of any broadband high-resolution study of the luminescence spectra of RE-doped crystals, as well as of a resolved hyperfine structure in these spectra. However, the luminescence spectra open up additional possibilities, such as, e.g., the study of very small and/or highly diluted samples or the development of new types of remote sensors. Here, we present the results of a high-resolution study of broadband PL spectra of LiYF_4_:Ho crystals in a magnetic field.

## Results

### Photoluminescence of LiYF_4_:Ho^3+^ in zero magnetic field: hyperfine structure

**Fig. 1:**
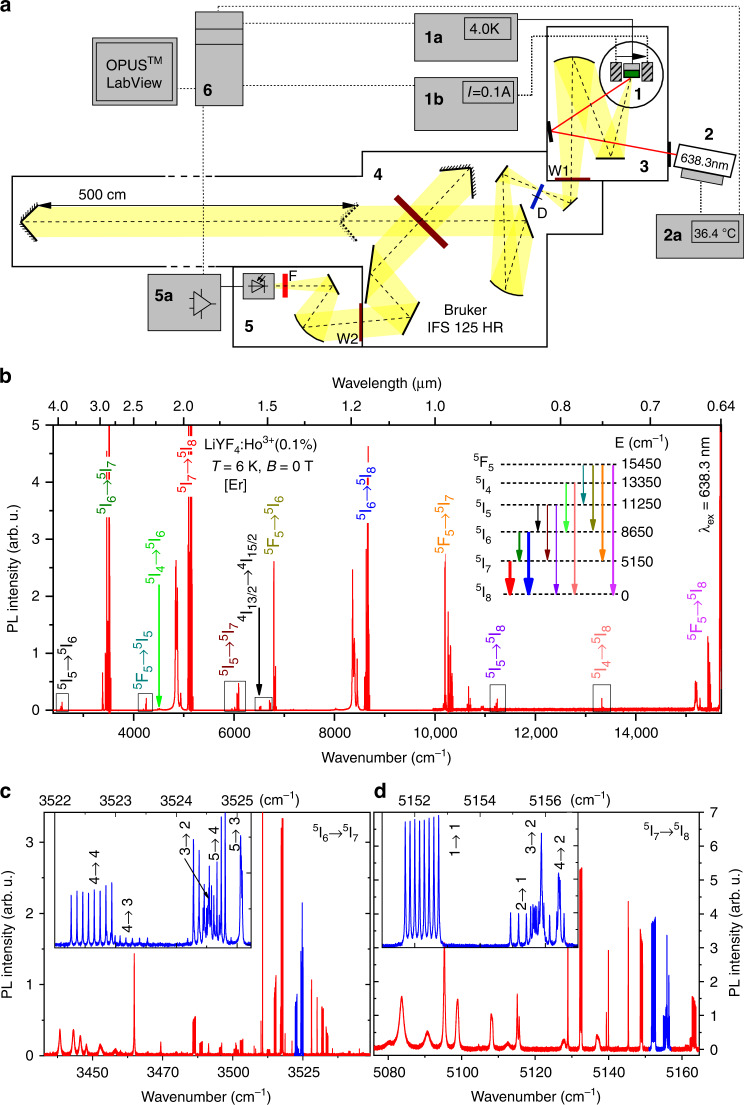
PL spectrum of LiYF_4_:Ho^3+^ (0.1 at. %): *T* = 6 K, *B* = 0; λ_ex_ = 638.3 nm. **a** Setup: 1—cryostat with magnetic coils (gray hatched), 1a—temperature controller, 1b—current source for magnetic coils; 2—temperature-stabilized diode laser, 2a—temperature controller and current source for laser; 3—PL module; 4—Fourier spectrometer; 5—PL registration module, 5a—preamplifier and an analog to digital converter; 6—workstation for spectra calculation and automatic control of magnetic field and temperature. **b** The whole spectral region studied. Identified intermultiplet transitions of Ho^3+^ as well as the ^4^I_13/2_ → ^4^I_15/2_ transition of Er^3+^ trace impurity are indicated. Inset presents the scheme of energy levels of Ho^3+^; the observed transitions are shown by arrows. **c** ^5^I_6_ → ^5^I_7_ and **d** ^5^I_7_ → ^5^I_8_ luminescent transitions. Insets show the spectral lines highlighted in blue on an enlarged scale. The numbers n_i_ → n_f_ indicate the initial and final levels of the corresponding transition, numbered from the lowest level in the CF multiplet (Table 1).

Lithium yttrium fluoride crystals, LiYF_4_, exhibit the luminescence from many optically excited levels of RE ions doped into this crystal, in a wide spectral range. PL of a LiYF_4_:Ho^3+^ (0.1 at. %) crystal was excited by the wavelength 638.3 nm of a diode laser. This excitation wavelength corresponds to the transition from the ground state to the upper crystal-field (CF) level of the ^5^F_5_ multiplet of the Ho^3+^ ion (see Supplementary Fig. S1). High-resolution PL spectra were acquired on an experimental setup built on the basis of a Bruker IFS 125HR high-resolution Fourier spectrometer (Fig. 1a and Methods).

Figure 1b shows the low-temperature PL spectrum of LiYF_4_:Ho^3+^ (0.1 at. %), in the whole spectral range studied. The observed optical manifolds are identified in accordance with the scheme given in the inset. Figure 1c, d presents the spectral manifolds ^5^I_6_ → ^5^I_7_ and ^5^I_7_ → ^5^I_8_, respectively, in extended scales as examples. At low temperatures, only lowest-energy levels of the ^5^I_6_ and ^5^I_7_ excited CF multiplets are populated after the laser excitation. The lines in the low-frequency part of the luminescent manifolds correspond to transitions to the upper levels of the final CF multiplets, broadened due to phonon relaxation to underlying levels^9^. In the high-frequency parts of the manifolds, many lines with well-resolved hyperfine structure are observed, as insets of Fig. 1c, d show.

**Fig. 2:**
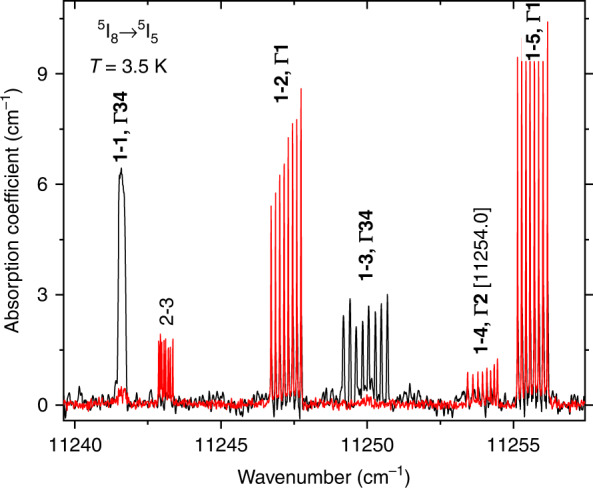
^5^I_5_ CF levels with resolved HFS. The π- (**k**⊥*c*, **E||***c*; black trace) and σ- (**k**⊥*c*, **E**⊥*c*; red trace) polarized absorption spectra of a LiYF_4_:Ho^3+^ (0.1 at. %) single crystal at the temperature 3.5 K in zero magnetic field. *δσ* = 1/2 *L* = 0.01 cm^−1^ (*L* is the maximal displacement of a moving mirror in the Fourier spectrometer).

The resolved hyperfine structure in optical absorption spectra of LiYF_4_:Ho^3+^ was discovered^9^ and studied in detail^9–12^ earlier. The CF energy levels of Ho^3+^ are characterized by the one-dimensional Γ_1_ and Γ_2_ and two-dimensional Γ_34_ irreducible representations (IR) of the *S*_4_ point symmetry group of the holmium site in LiYF_4_. Table 1 lists the energies and symmetries (IRs) of several CF levels of Ho^3+^ in LiYF_4_ (relevant for further discussion), determined from the analysis of polarized absorption and luminescence spectra, using the selection rules^11,12^ (see Supplementary Table S1). An extended table of CF levels is presented in Supplementary Table S3. The data obtained are consistent with the results of previous studies^9–12^, with one exception. Namely, we did not find the level 11,242.4 cm^−1^ (^5^I_5_)^12^, but demonstrated the level 11,254.0 cm^−1^, which is absent in ref. ^12^. Figure 2 illustrates this. We note that the allowed ED transition ^5^I_8_ (1, Γ_34_) → ^5^I_5_ (4, Γ_2_) [11,254 cm^−^^1^] is nevertheless very weak and can easily be missed if for some reason (higher holmium concentration, insufficient spectral resolution) the spectral lines are broadened.

**Table 1:** Information on CF levels of Ho^3+^ in LiYF_4_:Ho^3+^.

	*n*	IR	*E* (cm^−1^)	Δ_HFS_ (cm^−1^)	*g*_||_
^5^F_5_	3	Γ_1_	15,512.7		
	2	Γ_34_	15,495.4	0.034	-4.2
	1	Γ_2_	15,489.4		
^5^I_5_	5	Γ_1_	11,255.6		
	4	Γ_2_	11,254.0		
	3	Γ_34_	11,249.9	0.069	-2.8
	2	Γ_1_	11,247.2		
	1	Γ_34_	11,241.6	0.178	8.1
^5^I_6_	6	Γ_1_	8697.4		
	5	Γ_2_	8687.75		
	4	Γ_34_	8685.9	0.095	6.4
	3	Γ_34_	8680.3	0.015	1.0
	2	Γ_1_	8673.4		
	1	Γ_2_	8670.9		
^5^I_7_	6	Γ_1_	5206.1		
	5	Γ_34_	5184.7	0.131	−10.5
	4	Γ_2_	5163.3		
	3	Γ_1_	5162.8		
	2	Γ_34_	5155.75	0.088	6.8
	1	Γ_2_	5152.3		
^5^I_8_	3	Γ_2_	23.3		
	2	Γ_2_	6.85		
	1	Γ_34_	0	0.147	−13.3

Irreducible representations IRs, crystal-field energies *E* (cm^−1^), average intervals of the magnetic hyperfine structure Δ_HFS_ (cm^−1^), and *g* factors of Γ_34_ doublets, obtained from the analysis of the optical spectra of LiYF_4_:Ho^3+^ (0.1 at. %).

In a zero magnetic field, the Γ_34_ CF levels possess an eight-component equidistant magnetic hyperfine structure resulting from the interaction of 4f electrons with the magnetic moment of the holmium nucleus with spin *I* = 7/2. Each hyperfine component is doubly degenerate, the states |Γ_3_, *m*> and |Γ_4_, –*m*> have the same energy (here, *m* is the component of nuclear moment ***I*** along the crystallographic *c* axis, –7/2 ≤ *m* ≤ 7/2)^9,12^. For the Γ_1_ and Γ_2_ non-degenerate electronic CF states magnetic HFS is forbidden in the first approximation. Electric quadrupole and pseudoquadrupole (magnetic dipole in the second approximation) hyperfine interactions split Γ_1_ and Γ_2_ singlets into four nonequidistant hyperfine sublevels and lead to nonequidistance in Γ_34_ hyperfine manifolds^10^.

In the luminescence spectra, HFS is observed in other spectral regions compared to absorption spectra, in particular, at telecommunication wavelengths around 1.5 μm (transitions ^5^F_5_ → ^5^I_6_ and ^5^I_5_ → ^5^I_7_), which can be used, for example, to implement remote magnetic field sensors. For sensor applications, such parameters as the luminescence linewidth and sensitivity of the line position to perturbations are important.

### Isotopic structure in the photoluminescence spectra of ^7^Li_1–*x*_^6^Li_*x*_YF_4_:Ho^3+^ and linewidths of hyperfine components

**Fig. 3:**
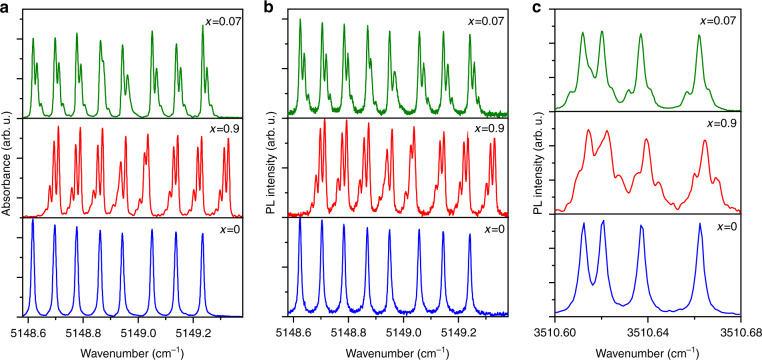
Isotopic structure of hyperfine components in the spectra of crystals with different ^6^Li and ^7^Li isotope compositions. Isotopic structure in the **a** absorption and **b**, **c** luminescence spectra of ^7^Li_1–*x*_^6^Li_*x*_YF_4_:Ho^3+^ (0.1 at %) crystals with *x* = 0.07 (natural abundance of Li isotopes; green traces), *x* = 0.9 (red traces), and *x* = 0 (blue traces). **a** ^5^I_8_ Г_2_ (6.85) → ^5^I_7_ Г_34_ (5155.75), **b** ^5^I_7_ Г_34_ (5155.75) → ^5^I_8_ Г_2_ (6.85), and **c** ^5^I_6_ Г_1_ (8673.4) → ^5^I_7_ Г_1_ (5162.8) optical transitions. *T* = 6 K. *δσ* = 1/2 *L* = 0.001 cm^−1^ (*L* is the maximal displacement of a moving mirror in the Fourier spectrometer).

Figure 3a, b shows the absorption and PL spectra, respectively, of ^7^Li_1–*x*_^6^Li_*x*_YF_4_:Ho^3+^ single crystals with different lithium isotope compositions, in the region of the optical transition between the ^5^I_7_ Г_34_ (5155.7 cm^−1^) and ^5^I_8_ Г_2_ (6.85 cm^−1^) CF levels of Ho^3+^. An identical fine structure of the hyperfine components is clearly observed in the absorption and PL spectra if *x* ≠ 0. Earlier, the structure observed in the absorption spectra was unambiguously ascribed to the isotopic disorder in the lithium sublattice^26,27^. Because of the difference in masses of the lithium isotopes, the amplitudes of their zero-field vibrations differ, leading to different equilibrium positions of the nearest fluorine ions (through the anharmonicity of vibrations) and, thus, to the dependence of the crystal field for the Ho^3+^ ion on the lithium isotope composition in its nearest surrounding^26^. Evidently, the origin of the fine structure of the hyperfine components in the PL spectra is the same. Figure 3c gives an example of such isotopic structure in the PL line corresponding to a singlet–singlet transition.

In what follows, we study PL spectra of LiYF_4_:Ho^3+^ in an external magnetic field. To avoid complexities associated with the isotopic structure, we use a monoisotopic ^7^LiYF_4_:Ho^3+^ crystal. Preliminarily we investigated the widths of the PL lines in a zero magnetic field. Here, a remark is necessary concerning the resolution. The instrumental function of a Fourier spectrometer has the form $f_L(\sigma ) = \frac{{\sin x}}{x}$, $x = 4\pi L\sigma$, where *σ* is the wavenumber and *L* is the maximal displacement of a moving mirror in a Michelson interferometer. The distance between the first zeros of *f*_*L*_(*σ*) equals *δσ* = 1/2 *L* (0.001 cm^−1^ in our case) and is indicated as “resolution” in an instrument manual. The full width at half maximum (FWHM) of *f*_*L*_ is 0.6*δσ*. The measured line shape is a convolution of a real line shape and the instrumental function *f*_*L*_. The line shape practically does not change down to *W* = 2*δσ* (*W* is FWHM of the studied spectral line). At *W* = *δσ*, weak additional maxima appear on the line wings, and in the case of the Loretzian line shape, FWHM of the convolution exceeds by 10% FWHM of the original line^34^ (see Supplementary Fig. S3).

**Fig. 4:**
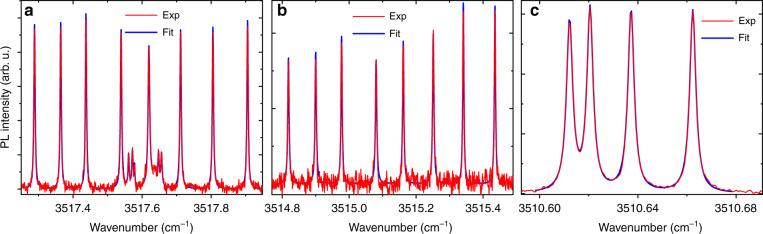
Hyperfine structure of several photoluminescence lines in the ^5^I_6_ → ^5^I_7_ transition of Ho^3+^:^7^LiYF_4_ at *T* = 6 K. Lines correspond to the transitions **a** 2 → 2 [^5^I_6_ Г_1_ (8673.4) → ^5^I_7_ Г_34_ (5155.75)] and 3 → 3 [^5^I_6_ Г_34_ (8680.3) → ^5^I_7_ Г_1_ (5162.8)]; **b** 1 → 2 [^5^I_6_ Г_2_ (8670.9) → ^5^I_7_ Г_34_ (5155.75)]; **c** 2 → 3 [^5^I_6_ Г_1_ (8673.4) → ^5^I_7_ Г_1_ (5162.8)]. Red traces are experimental spectra registered with *δσ* = 0.001 cm^−1^, blue curves represent the results of fit with sets of Loretzians. Note the stretched wavenumber scale at **c**.

Figure 4 shows several PL lines of the ^5^I_6_ → ^5^I_7_ transition in Ho^3+^:^7^LiYF_4_. The lines represented in Fig. 4a, b correspond to the transitions from the lowest in the ^5^I_6_ multiplet Г_1_ and Г_2_ CF singlets, respectively, to the ^5^I_7_ Г_34_ (5155.75 cm^−1^) doublet and reflect HFS of the latter. The four-component line of Fig. 4c originates from a singlet–singlet transition.

To find the correct linewidths of the hyperfine components, we approximated the observed spectra with sets of Loretzians. The FWHM thus obtained range from 0.002 to 0.004 cm^−1^ (for comparison, the smallest linewidth observed in the absorption spectra of this crystal is 0.012 cm^−1^). The ^5^I_6_ → ^5^I_7_ transitions, inaccessible in absorption, exhibit the narrowest spectral lines. The PL linewidth of 0.002 cm^−1^ observed here is, as far as we know, the smallest inhomogeneous linewidth ever observed in the luminescence spectra of crystals.

### Photoluminescence of ^7^LiYF_4_:Ho^3+^ in an external magnetic field *B*||*c*

**Fig. 5:**
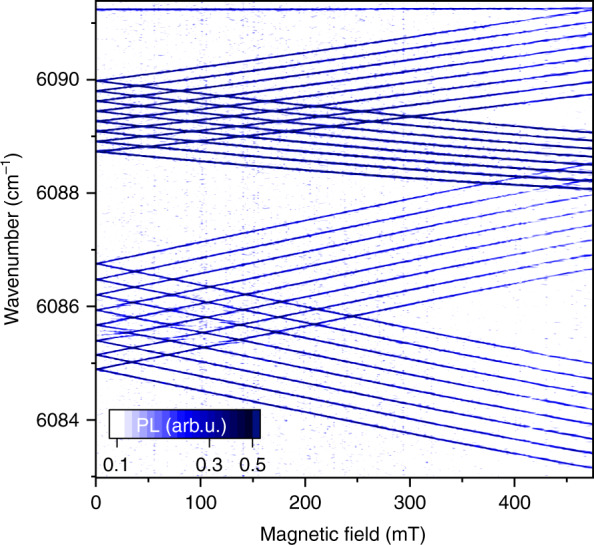
Evolution of the hyperfine structure with increasing magnetic field *B*||*c*. Photoluminescence intensity map in the magnetic-field – wavenumber scale for two transitions from the ^5^I_5_ Г_34_ (11,241.6) CF level to the levels of the ^5^I_7_ CF multiplet: ^5^I_7_ Г_2_ (5152.3) (top line) and ^5^I_7_ Г_34_ (5155.75) (bottom line). ^7^LiYF_4_:Ho^3+^ (0.1 at. %), *T* = 10 K. ***B***||*c*.

The twofold degeneracy of the hyperfine states |Γ_3_, *m*> and |Γ_4_, –*m*> is lifted in an external magnetic field parallel to the *c* axis of the crystal, ***B***||*c*. The electronic Zeeman energies are equal to ± *g*_||_*μ*_B_*B*/2, where *μ*_B_ = 0.4669 cm^−1^T^−^^1^ is the Bohr magneton and *g*_||_ is the *g* factor of a doublet. Figure 5 shows HFS in a magnetic field ***B***||*c* for two PL lines of ^7^LiYF_4_:Ho^3+^ (0.1 at. %) starting from the ^5^I_5_ Г_34_ doublet at 11,241.6 cm^−1^. The line 6089.3 cm^−1^ has the ^5^I_7_ Г_2_ singlet at 5152.3 cm^−1^ as a terminal level, its splitting is governed by the *g* factor *g*_||_ = 8.1 of the ^5^I_5_ Г_34_ (11,241.6) doublet. Magnetic *g* factors of several Г_34_ doublets given in Table 1 were determined experimentally by analyzing the splitting of PL lines corresponding to transitions between a given doublet and a singlet. We note that the *g* factor is proportional to the hyperfine interval Δ_HFS_ (ref. ^9^): | *g*_||_ | = 2*g*_0_Δ_HFS_ /*A*_J_, where *g*_0_ is the Lande factor and *A*_J_ is the magnetic hyperfine constant (see Supplementary information, Eqs. S5, S9, and S10).

The line 6085.85 cm^−1^ ends at the ^5^I_7_ Г_34_ (5155.75) doublet, and the hyperfine interval in HFS of this line is a sum of the hyperfine intervals in the initial ^5^I_5_ Г_34_ (11,241.6) and final ^5^I_7_ Г_34_ (5155.75) doublets of the transition but the splitting follows the sum of *g* factors of the involved levels, *g*_||_(11,241.6) + *g*_||_(5155.75) = 8.1 + 6.8 = 14.9 (see Supplementary information for more details; Supplementary Figs. S4–S34 show the magnetic-field-dependent PL spectra used to obtain the data given in Table 1).

### Hyperfine levels’ anticrossing in the photoluminescence spectra of ^7^LiYF_4_:Ho^3+^ in a magnetic field *B*||*c*

**Fig. 6:**
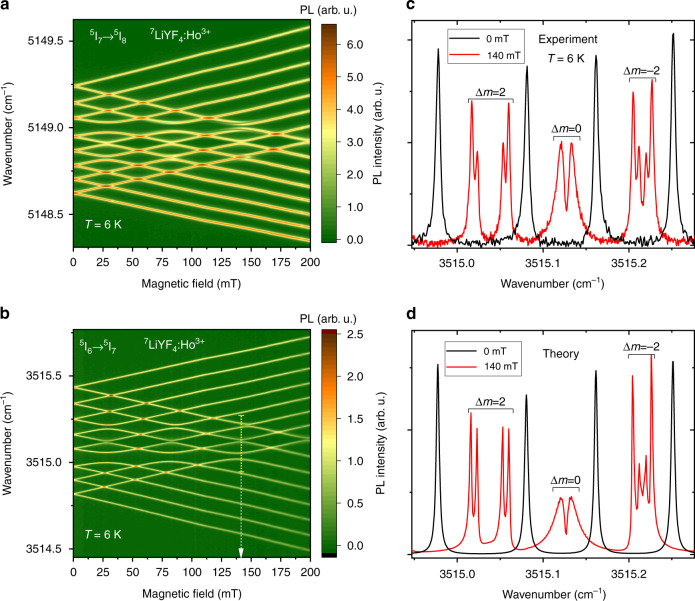
Anticrossings of the hyperfine levels in the luminescence spectra of ^7^LiYF_4_:Ho^3+^ in a magnetic field *B*||*c*. **a**, **b** Luminescence intensity maps in the magnetic-field – wavenumber scale for the **a** ^5^I_7_ Г_34_ (5155.75) → ^5^I_8_ Г_2_ (6.85) and **b** ^5^I_6_ Г_2_ (8670.9) → ^5^I_7_ Г_34_ (5155.75) transitions in ^7^LiYF_4_:Ho^3+^ (0.1 at. %) at 6 K. Anticrossings of the hyperfine levels are observed. **c** Experimental and **d** simulated fragment of the spectrum corresponding to the ^5^I_6_ Г_2_ (8670.9) → ^5^I_7_ Г_34_ (5155.75) transition of ^7^LiYF_4_:Ho^3+^ (0.1 at. %) in a zero magnetic field (in black) and in the field of 140 mT (in red), indicated by an arrow in **b**.

Figure 6a presents the PL intensity map for the radiative transition from the lowest electronic doublet in the ^5^I_7_ CF multiplet, ^5^I_7_ Г_34_ (5155.75 cm^−1^), to the first excited singlet of the ground ^5^I_8_ CF multiplet, ^5^I_8_ Г_2_ (6.85 cm^−1^). The presented picture coincides with the one observed in the absorption spectra at the corresponding transition^33^ and demonstrates gaps at the anticrossings of the |Γ_4_, *m*> and |Γ_3_, *m* ± 2> states (|Δ*m*| = 2) (see, e.g., a “huge” gap at 5149.0 cm^−1^ in Fig. 6a) and of the |Γ_4_, *m*> and |Γ_3_, *m*> states (|Δ*m*| = 0). However, it should be noted that when measuring luminescence, a better contrast is achieved.

Narrowest PL lines of ^7^LiYF_4_:Ho^3+^ (0.1 at. %) in a magnetic field were observed in the case of the ^5^I_6_ → ^5^I_7_ transitions, the same as in a zero magnetic field. Figure 6b shows a representative example. Here, the spectrum of the ^5^I_6_ Г_2_ (8670.9) → ^5^I_7_ Г_34_ (5155.75) transition is shown. This transition in the spectral region of 2.84 μm involves the same ^5^I_7_ Г_34_ (5155.75) CF doublet as the transition at 1.94 μm presented in Fig. 6a. Although the singlets participating in the transitions [^5^I_8_ Г_2_ (6.85 cm^−1^) in the case of Fig. 6a and ^5^I_6_ Г_2_ (8670.9) in the case of Fig. 6b] are different, the spectra are similar.

Figure 6c shows a fragment of the spectrum corresponding to the ^5^I_6_ Г_2_ (8670.9) → ^5^I_7_ Г_34_ (5155.75) transition of ^7^LiYF_4_:Ho^3+^ (0.1 at. %) in a zero magnetic field and in the field of 140 mT at one of the anticrossings. A four-component structure at the |Δ*m*| = 2 anticrossings and a very specific line shape at the Δ*m* = 0 anticrossing are observed.

**Fig. 7:**
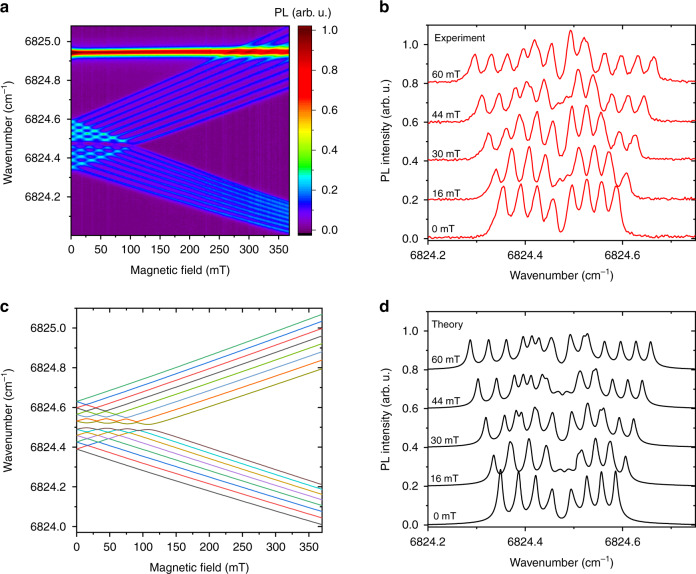
The ^5^F_5_ Г_34_ (15,495.4) level demonstrates the largest deformation splitting. **a** Luminescence intensity map in the magnetic-field – wavenumber scale and **b** calculated frequency vs magnetic field dependences for the ^5^F_5_ Г_34_ (15495.4) → ^5^I_6_ Г_2_ (8670.9) transition in ^7^LiYF_4_:Ho^3+^ (0.1 at. %) at 10 K**. c** Experimental and **d** calculated spectra of this transitions at several values of the magnetic field. A strong horizontal line in **a** corresponds to the singlet–singlet transition ^5^F_5_ Г_1_ (15,512.7) → ^5^I_6_ Г_2_ (8687.75).

The ^5^F_5_ Г_34_ (15,495.4) CF level demonstrates the largest Δ*m* = 0 gaps, which are observed in all the lines starting from this level. As an example, Fig. 7a shows the spectrum of the ^5^F_5_ Г_34_ (15,495.4) → ^5^I_6_ Г_2_ (8670.9) transition. A large central gap is clearly seen in the hyperfine pattern in a magnetic field.

## Discussion

Luminescence spectra complement absorption spectra, providing information on levels inaccessible to absorption. In the case of spectra consisting of narrow lines, the signal-to-noise ratio in PL spectra is much higher than in absorption spectra. Moreover, in some cases, the PL lines are noticeably narrower than the absorption ones, so that the HFS is resolved much better. A good example can be found in Fig. 4, where, in particular, quadrupole HFS of a Γ_1_ → Γ_1_ magnetic dipole transition is observed (Fig. 4c, see Supplementary Table S1 for selection rules). The use of luminescence instead of absorption makes it possible to work with micron- or even nano-sized samples, which is important, e.g., for sensor applications. The PL spectra of LiYF_4_:Ho^3+^ can be used to control the external magnetic field. Here, the ^5^F_5_ → ^5^I_6_ and ^5^I_5_ → ^5^I_7_ luminescent transitions near 1.64 and 1.46 μm, respectively, fall into the transparency window of optical fibers and offer a possibility to implement, for example, remote magnetic field sensors. Let us consider the PL line at 6085.85 cm^−1^ [^5^I_5_ Г_34_ (11241.6) → ^5^I_7_ Г_34_ (5155.75), see Fig. 5], the magnetic-field splitting of which, Δ = *μ*_B_ *g*_||_ *B* (see Supplementary Eq. S4), follows the sum of *g* factors of the levels involved: *g*_||_ = *g*_||_(11,241.6) + *g*_||_(5155.75) = 14.9. The position of a spectral line with the known shape can be determined with an accuracy δσ equal to 0.1 FWHM of the instrumental function^34^ (in Fourier spectroscopy, where the wavenumber scale over the entire spectral region is set by a stabilized He-Ne laser, this is also the accuracy of the absolute wavenumber scale^35^). In our case, the instrumental function is characterized by FWHM = 0.6/2 *L*. For *L* = 500 cm we find δσ = 0.6·10^−^^4^ cm^−1^. Using the relation δΔ = 2δσ = *μ*_B_*g*_||_δ*B*, one obtains δ*B* = 17 μT, i.e., the strength of an external magnetic field can be determined with the precision δ*B* ≈ 17 μT. It is worth noting that the range of measured magnetic fields can be very broad (calibration curve σ(*B*) can be used for strong fields, for which Supplementary Eq. S4 is not valid). It is also possible to determine the direction of the magnetic field by installing two LiYF_4_:Ho crystals with the mutually perpendicular *c* axes (*g*_⊥_ = 0 for non-Kramers doublets). To control small deviations of the magnetic field strength, the method developed for magnetometry with nitrogen-vacancy (NV) centers in diamond^36^ can be applied. In ref. ^36^, a field-dependent position of a dip in the NV luminescence intensity was controlled with an accuracy of ~μT using AOM-modulated laser excitation and a lock-in amplifier. The authors of ref. ^36^ noted that their “technique can be extended to other magnetically sensitive features in the NV PL or absorption, as well as features associated with other spin defects in solid-state systems”. In particular, a narrow hyperfine component in the luminescence spectrum of ^7^LiYF_4_:Ho^3+^ can be used instead of a dip in a broad luminescence spectrum of NV. Unlike the case of NV centers^36^, no additional field (102.4 mT, to set at the ground-state level anticrossing, and a secondary coil to apply small modulation of this field) is necessary in the case of LiYF_4_:Ho, which can be favorable for quantum technology devices.

Similar to a ruby luminescent pressure sensor, a small piece of LiYF_4_:Ho crystal can be placed nearby a quantum-memory crystal in the repeater, operating at low temperatures, to control an external magnetic field used to adjust the energy levels. However, the implementation of a practical and convenient sensor requires additional work. A high-resolution Fourier spectrometer, being the only spectral instrument able to deliver information on the HFS in a wide spectral range, is a bulky and very expensive device. A possible solution to the problem is to isolate the desired spectral line with an interference filter and use a Fabry–Perot interferometer. To avoid overlay of the interference orders, the free spectral range should be 2 cm^−1^, which dictates a distance between Fabry–Perot mirrors 2.5 mm. In order to obtain a resolution of about 0.002 cm^−1^ in this case, it is necessary to have a finesse of about 1000. The finesse depends on the reflection coefficient of the mirrors and the quality of their surface. Currently, firms offer Fabry–Perot interferometers with finesse up to 1500 in a wide spectral range^37^.

Monoisotopic crystals are most favorable for realizing sensitive sensors, because they demonstrate the narrowest lines (as Fig. 3 convincingly shows). On the other hand, the isotopic structure in the spectra of LiYF_4_:Ho^3+^ reflects the lithium isotope composition in a crystal. Under neutron irradiation, the ^6^Li isotopes can capture neutrons converting to ^7^Li. A change of the isotope composition in a crystal originally enriched with the ^6^Li isotope could be detected by PL spectra.

Next, we turn to the anticrossings observed in the PL spectra. As was shown in ref. ^33^ on the basis of magnetic-field-dependent absorption spectra of ^7^LiYF_4_:Ho^3+^, gaps observed at the anticrossings of the |Γ_4_, *m*> and |Γ_3_, *m* ± 2> states (|Δ*m*| = 2) are caused by the transverse term in magnetic dipole hyperfine interaction but the four-component structure at the |Δ*m*| = 2 anticrossings arises due to the mixing of the electron-nuclear wave functions of the crossing levels in the vicinity of the anticrossing point (at the anticrossing point, the wave functions of the crossing levels are present with equal weights). The Δ*m* = 0 gaps have a different nature, they are due to random crystal-lattice deformations always present in a real crystal^33^. Here, we focus on anticrossings of the hyperfine levels with equal nuclear spin projections, Δ*m* = 0, which can be used to check crystal quality. In particular, by analyzing PL of Ho^3+^ (either introduced in a small amount or present as uncontrolled impurity) in ^7^LiYF_4_:Nd, one can check the quality of this crystal proposed^7^ for a quantum memory based on off-resonant Raman interaction.

A very specific line shape with a sharp dip in the center and slopy wings is especially well seen just in the luminescence spectra (see Fig. 6c). Previously, such line shape was observed for electronic transitions between singlet and doublet CF levels in the spectra of LiYF_4_:Tm^3+^ (ref. ^23^), Cs_2_NaYF_6_:Yb^3+^ (ref. ^31^), and Tm^3+^ in ABO_4_ (A = Y, Lu; B = P, V)^32^ and was shown to be the result of random lattice deformations^23,31,32^. A theory has been developed that made it possible to extract information about the strain distribution function from the analysis of the line shape^32^. The measured shape of the PL line with a dip at the center in the region of the Δ*m* = 0 anticrossings for the singlet-doublet transition ^5^I_6_ Г_2_ (8670.9) → ^5^I_7_ Г_34_ (5155.7) is modeled here on the basis of this theory.

The Hamiltonian of the electronic 4f^10^ shell of the Ho^3+^ impurity ion, $H = H_{{{{\mathrm{FI}}}}} + H_{{{{\mathrm{CF}}}}} + V$, where $V = H_{{{{\mathrm{MHF}}}}} + H_{{{{\mathrm{QHF}}}}} + H_Z + H_{{{{\mathrm{ElD}}}}}$, contains the free-ion energy *H*_FI_, the interaction with the crystal-field *H*_CF_, the magnetic (*H*_MHF_) and quadrupole (*H*_QHF_) hyperfine interactions, the electronic and nuclear Zeeman energies *H*_Z_ in an external magnetic field, and the electron-deformation interaction *H*_ElD_, linear in components $e_{\alpha \beta }$ of the strain tensor ***e***. Modeling of the spectral profiles involves consequent numerical diagonalizations of the Hamiltonian *H*_0_ = *H*_FI_ + *H*_CF_ defined in the total space of 1001 electronic states and, at the next step, of the projection of the operator *V* onto the truncated basis of 76 × 8 = 608 electron-nuclear states corresponding to the lower 76 eigenvalues ($E_{\it{\Gamma }}$) of the Hamiltonian *H*_0_. The obtained energies $E_{{\it{\Gamma }}j}$ and wave functions of the electron-nuclear sublevels of the ^5^I_*J*_ (*J* = 4,…8) and ^5^F_5_ multiplets are used to calculate intensity distributions in spectral lines corresponding to magnetic (electric) dipole radiative transitions ${\it{\Gamma }} \to {\it{\Gamma }}^\prime$ at fixed values of the magnetic field strength and strain tensor components,

1 $$I_{\Gamma \Gamma ^\prime }(E,{{{\boldsymbol{e}}}}) = \mathop {\sum}\limits_{j \in {\it{\Gamma }}} {\mathop {\sum}\limits_{k \in {\it{\Gamma }}^\prime } {\mathop {\sum}\limits_\alpha {\left| { \langle {\it{\Gamma }}^\prime k} \right|M_\alpha \left| {{\it{\Gamma }}j \rangle } \right|^2} } } I_{0,\Gamma \Gamma ^\prime }(E_{{\it{\Gamma }}j} - E_{{\it{\Gamma }}^\prime k} - E)$$

Here ***M*** is the magnetic (electric) dipole moment operator of ten 4f electrons and $I_{0,\Gamma \Gamma ^\prime }(x) = (\delta E_{\Gamma \Gamma ^\prime }/\pi )(x^2 + \delta E_{\Gamma \Gamma ^\prime }^2)^{ - 1}$ is the form-function with FWHM 2$\delta E_{\Gamma \Gamma ^\prime }$ of individual transitions between hyperfine sublevels of the CF states ${\it{\Gamma }}$and ${\it{\Gamma }}^\prime$. Note that for the intraconfigurational transitions, the structure of the effective even electric dipole moment operator is determined by the odd component of the crystal field. The finite spectral envelope is modeled by averaging the distribution (1) with the distribution function *g*(***e***) of random strains induced by lattice point defects in LiYF_4_ crystals, taking into account the elastic anisotropy of the crystal lattice^32^. The most pronounced spectral effect of random strains, namely, the formation of a dip in the envelope of the transition involving a CF non-Kramers doublet Γ_34_, is caused by rhombic strains $e(B_g^1) = (e_{xx} - e_{yy})/2$ and $e(B_g^2) = e_{xy}$, which transform according to the *B*_g_ IR of the *C*_4h_ lattice factor group and split Γ_34_ doublets. The corresponding distribution function can be written as follows:

2 $$g\left( {e\left( {B_g^1} \right),e\left( {B_g^2} \right)} \right) = \frac{{\nu _{B_g}\gamma _{B_g}}}{{2\pi }}\left( {\nu _{B_g}^2e_1^2 + e_2^2 + \gamma _{B_g}^2} \right)^{ - 3/2}$$

where $\nu _{B_g}$ = 2.44, $e_1 = e(B_g^1)\cos \varphi - e(B_g^2)\sin \varphi$, $e_2 = e(B_g^1)\sin \varphi + e(B_g^2)\cos \varphi$, $\varphi$ = 28.7° (ref. ^32^), and $\gamma _{B_g}$, which is proportional to the concentration of defects and determines the distribution width, is considered as a fitting parameter.

Experimental shapes of spectral lines were satisfactorily reproduced by computations of integrals over a two-dimensional space of random strains ($x = e(B_g^1),y = e( {B_g^2} )$)

3 $$I_{\Gamma \Gamma ^\prime }\left( E \right) \sim {\int\!\!\!\!\!\int} {I_{\Gamma \Gamma ^\prime }} (E,x,y)g(x,y)dxdy$$

where we used $\gamma _{B_g}$ = 5$\cdot$10^–5^ and parameters of the electron-deformation interaction presented in ref. ^33^. Examples of the obtained spectral envelopes in external magnetic fields (including fields with strengths corresponding to the Δ*m* = ±2 and Δ*m* = 0 anticrossings) are shown in Figs. 6d and 7d.

In summary, we have presented the first observation of the resolved hyperfine structure in the luminescence spectra of a RE-doped crystal and demonstrated PL lines as narrow as 0.002–0.004 cm^−1^ and with magnetic *g* factors as large as 10–15, including in the telecommunication spectral range. These PL lines are promising for creating remote magnetic field sensors that do not require an additional constant or variable magnetic field and/or microwave field and are capable of operating in a very wide range of measured magnetic fields. Our results pave the way for the development of a remote magnetic field sensor for, e.g., quantum repeaters installed in an extended quantum communication line. This work is also the first observation of hyperfine levels anticrossings in the luminescence spectra. The Δ*m* = 0 anticrossings can be used to evaluate random lattice deformations in crystals for quantum information devices, i.e., the crystal quality.

## Materials and methods

### Crystal growth

Three single crystals of LiYF_4_:Ho (0. 1 at. %) with different content of ^7^Li and ^6^Li isotopes were grown by the Stockbarger method. Li_2_CO_3_ with known lithium isotope composition was taken as a starting material. It was transformed into LiF by the “dry” method. The crystals were then grown from the mixture of appropriate fiuorides. Samples with dimensions 3 × 3 × 8 mm^3^ and containing the *c* axis in the 3 × 3 mm^2^ plane were cut from the x-ray oriented crystals and polished.

### Optical spectroscopy and low-temperature measurements

In the PL experiments displayed in Fig. 1a, a LiYF_4_:Ho^3+^ sample was placed into a cryomagnetic system (**1**) of our own design based on a Sumitomo RP096 closed-cycle helium cryostat. The design feature was that the electromagnet together with the concentrating magnetic system was attached directly to the first stage of the cryostat, which reduced the system dimensions, eliminated the need for heat removal in a vacuum, and allowed the use of higher currents and, consequently, magnetic fields, as the resistivity of copper winding wires was significantly reduced. A computer-controlled multi-channel current source Korad KA3305P (**1b**) was used to change the magnitude of the applied magnetic field. The magnetic field was directed along the *c* axis of the crystal and could be varied from 0 to 500 mT. The sample temperature was chosen such that several lower CF levels of each excited CF multiplet were populated, but the spectral lines were not yet broadened due to the electron–phonon interaction, and were set in the range from 3.5 to 10 K. The temperature was measured with a Si-diode temperature sensor (Scientific Instruments Si-410-AA) and a temperature controller (Lakeshore 335) (**1a**) and was stabilized with the precision of ±0.05 K during the entire measurement period. Emission was excited by the linearly polarized light of a diode laser (Oclaro HL63193) with 100 mW power, incident perpendicular to the *c* axis of the crystal and polarized along the *c* axis; the spot on the crystal was 0.5 mm. The laser wavelength could be changed in the interval 635 ± 10 nm by changing the temperature in the range from *t* = –20 to +45 °C using a self-made thermoelectric cooler and resistive heater. The laser temperature +36.4 °C was chosen by searching for the maximum PL signal. A homemade temperature stabilization system with feedback (**2a**) based on a temperature controller Scientific Instruments M9700 was implemented. Thus selected laser wavelength 638.3 nm excited the upper CF level of the ^5^F_5_ multiplet (Supplementary Fig. S1). Emission from the crystal was collimated by a mirror (diameter 90 mm, focal length 418 mm) in a homemade evacuated (10^–6^ Torr) PL module (**3**) separated from a Fourier spectrometer (**4**) by a CaF_2_ window W1. This gave a possibility of recording the spectra in the region of the atmospheric absorption in the middle and far infrared. Emission spectra were registered on a Bruker IFS 125HR high-resolution vacuum Fourier spectrometer (**4**) in the wavenumber range 2500–16,000 cm^−1^, with the maximal displacement of a moving mirror in the Michelson interferometer up to *L* = 500 cm, which provided the instrumental function with FWHM = 0.6 *δσ*, where *δσ* = 1/2 *L* = 0.001 cm^−1^ (see Supplementary Eq. S2). Thus, the minimal width of the instrumental function used to register PL spectra was 0.0006 cm^−1^ (18 MHz). The smallest input diaphragm D used was 0.3 mm in diameter. Polarized PL spectra were measured using a BaF_2_ crystal-based polarizer for the mid- and near-infrared ranges. Radiation from the output of the Michelson interferometer was directed to a homemade registration module (**5**) separated from the spectrometer by a CaF_2_ window W2, with an optical cutoff filter F to eliminate excitation laser light [Semrock single notch filter NF03-633E or a germanium plate (red in Fig. 1a)]. Depending on the studied region of the spectrum, highly sensitive detectors based on InSb (1800–5500 cm^−1^), high-gain InGaAs (5500–9000 cm^−1^), or SiPM (9000–15,500 cm^−1^) semiconductors were used. The signal was amplified by preamplifiers of our own design and then fed to the 16-bit ADC of the spectrometer (**5a**). The spectrum was calculated as a Fourier transform of the interferogram on a workstation (**6**) equipped with Bruker OPUS^TM^ software, LabView^TM^ software, and homemade software modules for COM ports that monitor and change sample and diode laser temperatures and electromagnet current. Polarized absorption spectra in zero magnetic field were acquired on the same experimental setup.

## Supplementary information

Supplementary Information

## Data Availability

The data that support the plots within the paper and other findings of this study are available from the corresponding author upon reasonable request.
