# Supplementary material for: Observation of the hyperfine structure and anticrossings of hyperfine levels in the luminescence spectra of LiYF4:Ho3+
Source: Light Sci Appl. 2022 Aug 2;11:245. doi: 10.1038/s41377-022-00933-2 (PMC9345886; doi:10.1038/s41377-022-00933-2)
Supplement: Supplementary file 1 — Supplementary Information [file 41377_2022_933_MOESM1_ESM.pdf]

Supplementary Information for

Observation of the hyperfine structure and anticrossings of hyperfine levels in the luminescence spectra of $LiYF_4:Ho^{3+}$

Kirill N. Boldyrev[1], Boris Z. Malkin[2], Marina N. Popova[1*]

[1]*Institute of Spectroscopy, Russian Academy of Sciences, Troitsk, Moscow, 108840 Russia*
[2]*Kazan Federal University, Kazan, 420008 Russia*

*Corresponding author popova@isan.troitsk.ru

---

1. The $^5I_8 \rightarrow {}^5F_5$ and $^5I_8 \rightarrow {}^5I_5$ optical multiplets (**Figs. S1, S2**)

2. Selection rules, number of crystal-field levels in *J* multiplets (**Tables S1, S2**)

3. Energy levels and observed transitions in optical spectra of $^7LiYF_4:Ho^{3+}$ (**Tables S3, S4**)

4. Convolution of the Lorentzian line shape with the instrumental function of a Fourier spectrometer (**Fig. S3**)

5. Magnetic *g* factors and their relationship with intervals in the magnetic hyperfine structure

6. PL spectra and PL intensity maps for different observed transitions. Magnetic *g* factors (**Figs. S4 – S34**)

1. The $^5I_8 \rightarrow {}^5F_5$ and $^5I_8 \rightarrow {}^5I_5$ optical multiplets

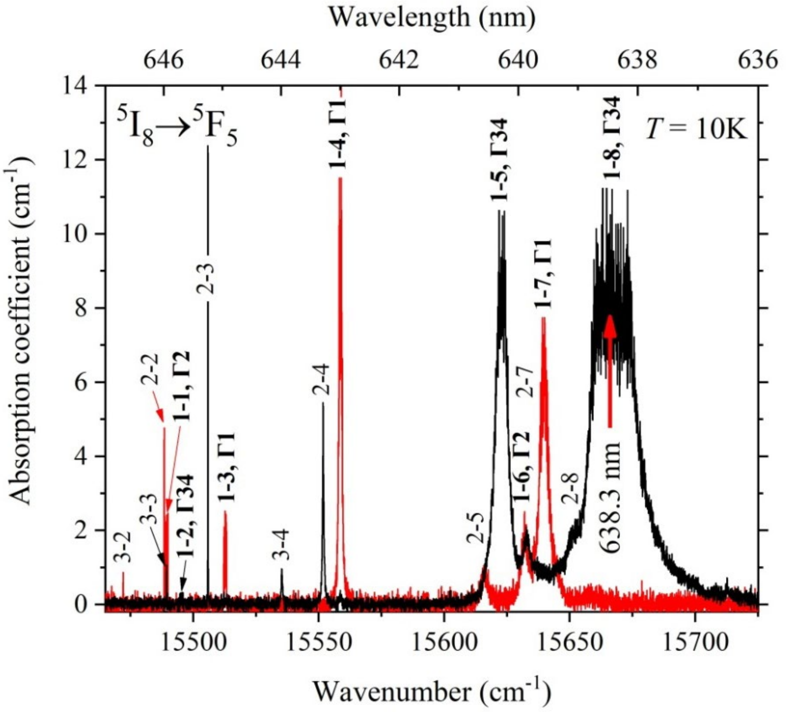

a

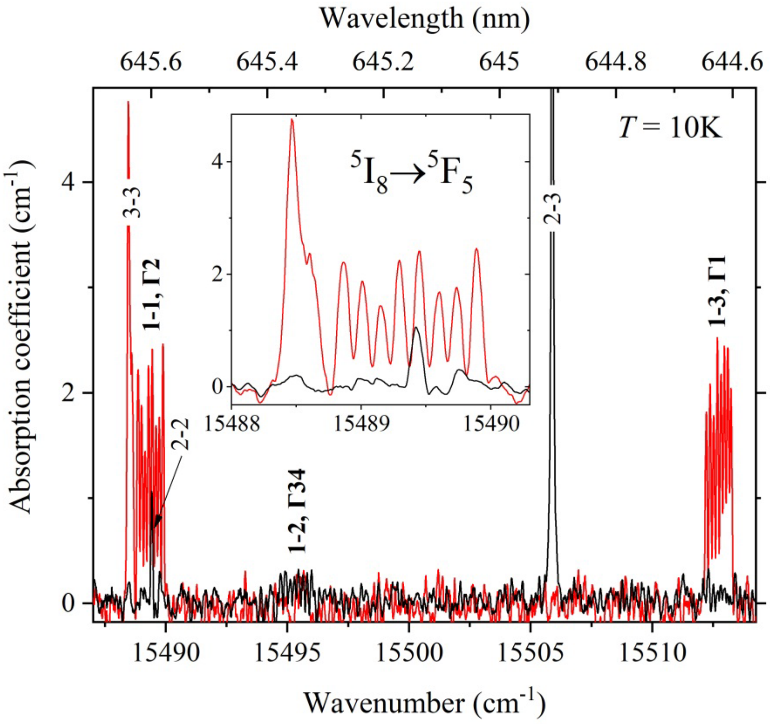

b

Figure S1. The π- (black trace) and σ- (red trace) polarized absorption spectra of a $LiYF_4{:}Ho^{3+}$ (0.1 at. %) single crystal at the temperature 10 K in a zero magnetic field. **a** The whole region of the $^5I_8 \rightarrow {}^5F_5$ optical multiplet and **b** the low-frequency part with resolved hyperfine structure. Lines originating from the $^5I_8$ (m) $\rightarrow$ $^5F_5$ (n) transitions are marked as m – n, where m (n) number the crystal-field levels of the $^5I_8$ ($^5F_5$) CF multiplets, starting from the lowest CF level (see Table S3). $\delta\sigma = 1/2L = 0.01$ cm^{-1} (L is the maximal displacement of a moving mirror in the Fourier spectrometer) The red arrow indicates the laser wavelength 638.3 nm used to excite the luminescence.

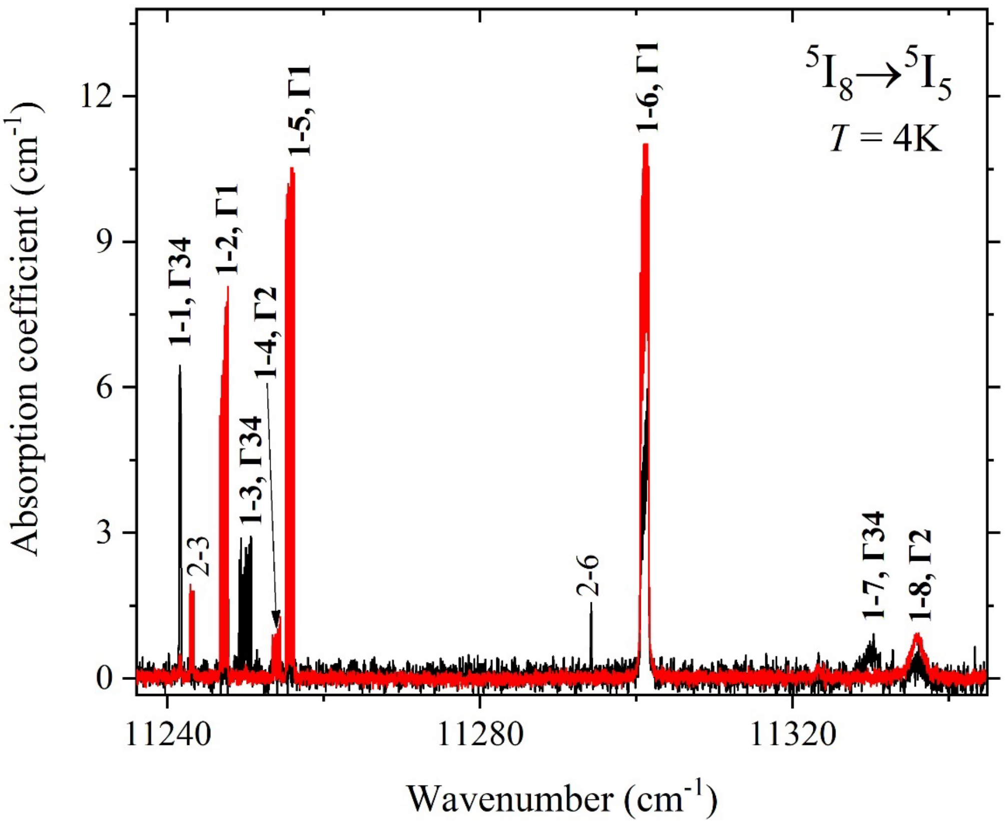

Figure S2. The π- (black trace) and σ- (red trace) polarized absorption spectra of a $LiYF_4:Ho^{3+}$ (0.1 at. %) single crystal at the temperature 4 K in a zero magnetic field. The whole region of the $^5I_8 \rightarrow {}^5I_5$ optical multiplet. Lines originating from the $^5I_8$ (m) → $^5I_5$ (n) transitions are marked as m – n, where m (n) number the crystal-field levels of the $^5I_8$ ($^5I_5$) CF multiplets, starting from the lowest CF level (see Table S3). $\delta\sigma = 1/2L = 0.01$ cm^{-1} (L is the maximal displacement of a moving mirror in the Fourier spectrometer).

2. Selection rules, number of crystal-field levels in *J* multiplets

Table S1. Selection rules. d_k (m_k), k = x, y, z, denote the allowed components of the ED (MD) transitions. For convenience, also polarizations are indicated, e.g. $\sigma_e \pi_m$ means that the transition is ED allowed in the σ polarization (**k**⊥*c*, **E**⊥*c*) and MD allowed in the π polarization (**k**⊥*c*, **E**||*c*).

<table>
<tr><td>S_4</td><td>Γ_1</td><td>Γ_2</td><td>Γ_3</td><td>Γ_4</td></tr>
<tr><td rowspan="2">Γ_1</td><td>m_z
σ_m</td><td>d_z
π_e</td><td>d_{x-iy}, $m_{x+i,y}$</td><td>d_{x+iy}, $m_{x-i,y}$</td></tr>
<tr><td></td><td></td><td colspan="2">σ_e π_m</td></tr>
<tr><td rowspan="2">Γ_2</td><td>d_z</td><td>m_z</td><td>d_{x+iy}, $m_{x-i,y}$</td><td>d_{x-iy}, $m_{x+i,y}$</td></tr>
<tr><td></td><td></td><td colspan="2">σ_e π_m</td></tr>
<tr><td>Γ_3</td><td></td><td></td><td>m_z
σ_m</td><td>d_z
π_e</td></tr>
<tr><td>Γ_4</td><td></td><td></td><td>d_z
π_e</td><td>m_z
σ_m</td></tr>
</table>

Table S2. Number of CF levels originated from a level with a given value of the total moment *J* of a free ion with even number of electrons, placed into the S_4-symmetry position in a crystal, and corresponding irreducible representations of CF levels.

J	**Number of CF levels (S_4)**	**Irreducible representations**
8	13	$5\Gamma_1+4\Gamma_2+4\Gamma_{34}$
7	11	$3\Gamma_1+4\Gamma_2+4\Gamma_{34}$
6	10	$3\Gamma_1+4\Gamma_2+3\Gamma_{34}$
5	8	$3\Gamma_1+2\Gamma_2+3\Gamma_{34}$

3. Energy levels and observed transitions in optical spectra of $^7LiYF_4:Ho^{3+}$

Table S3. Irreducible representations IR, crystal-field energies E (cm^{-1}), average intervals of the magnetic hyperfine structure Δ_{HFS} (cm^{-1}), and g factors of Γ_{34} doublets, obtained from the analysis of the optical spectra of $LiYF_4:Ho^{3+}$ (0.1 at. %).

	n	IR	E (cm^{-1})	Δ_{HFS} (cm^{-1})	$g_{\parallel}$		n	IR	E (cm^{-1})	Δ_{HFS} (cm^{-1})	$g_{\parallel}$
	6	Γ_1	8697.4				8	Γ_{34}	15667.1		
$^5I_6$	5	Γ_2	8687.75			$^5F_5$	7	Γ_1	15639.4		
	4	Γ_{34}	8685.9	0.095	6.4		6	Γ_2	15632.1		
	3	Γ_{34}	8680.3	0.015	1.0		5	Γ_{34}	15622.8		
	2	Γ_1	8673.4				4	Γ_1	15558.7		
	1	Γ_2	8670.9				3	Γ_1	15512.7		
							2	Γ_{34}	15495.4	0.034	-4.2
	6	Γ_1	5206.1				1	Γ_2	15489.4		
$^5I_7$	5	Γ_{34}	5184.7	0.131	-10.5						
	4	Γ_2	5163.3				8	Γ_2	11335.9		
	3	Γ_1	5162.8			$^5I_5$	7	Γ_{34}	11330.0		
	2	Γ_{34}	5155.75	0.088	6.8		6	Γ_1	11301.0		
	1	Γ_2	5152.3				5	Γ_1	11255.6		
							4	Γ_2	11254.0		
	3	Γ_2	23.3				3	Γ_{34}	11249.9	0.069	-2.8
$^5I_8$	2	Γ_2	6.85				2	Γ_1	11247.2		
	1	Γ_{34}	0	0.147	-13.3		1	Γ_{34}	11241.6	0.178	8.1

Table S4. Identification of the observed spectral lines mentioned in the main text

	Short notation	Frequency (cm^{-1})	
Fig. 1c	4 → 4	3522.6	$^5I_6$ (4) Γ_{34} (8685.9) → $^5I_7$ (4) Γ_2 (5163.3)
	4 → 3	3523.1	$^5I_6$ (4) Γ_{34} (8685.9) → $^5I_7$ (3) Γ_2 (5162.8)
	3 → 2	3524.55	$^5I_6$ (3) Γ_{34} (8680.3) → $^5I_7$ (2) Γ_{34} (5155.75)
	5 → 4	3524.45	$^5I_6$ (5) Γ_{34} (8687.75) → $^5I_7$ (4) Γ_2 (5163.3)
	5 → 3	3524.95	$^5I_6$ (5) Γ_{34} (8687.75) → $^5I_7$ (3) Γ_2 (5162.8)
Fig. 1d	1 → 1	5152.3	$^5I_7$ (1) Γ_2 (5152.3) → $^5I_8$ (1) Γ_{34} (0)
	2 → 1	5155.75	$^5I_7$ (2) Γ_{34} (5155.75) → $^5I_8$ (1) Γ_{34} (0)
	3 → 2	5155.95	$^5I_7$ (3) Γ_1 (5162.8) → $^5I_8$ (2) Γ_2 (6.85)
	4 → 2	5156.45	$^5I_7$ (4) Γ_2 (5163.3) → $^5I_8$ (2) Γ_2 (6.85)
Fig. 2	1 → 1	11241.6	$^5I_8$ (1) Γ_{34} (0) → $^5I_5$ (1) Γ_{34} (11241.6)
	2 → 3	11243.05	$^5I_8$ (2) Γ_2 (6.85) → $^5I_5$ (3) Γ_{34} (11249.9)
	1 → 2	11247.2	$^5I_8$ (1) Γ_{34} (0) → $^5I_5$ (2) Γ_{34} (11247.2)
	1 → 3	11249.9	$^5I_8$ (1) Γ_{34} (0) → $^5I_5$ (3) Γ_{34} (11249.9)
	1 → 4	11254.0	$^5I_8$ (1) Γ_{34} (0) → $^5I_5$ (4) Γ_{34} (11254.0)
	1 → 5	11255.6	$^5I_8$ (1) Γ_{34} (0) → $^5I_5$ (5) Γ_{34} (11255.6)
Fig. 3a,b, 6a	2 ↔ 2	5148.9	$^5I_8$ (2) Γ_2 (6.85) ↔ $^5I_7$ (2) Γ_{34} (5155.75)
Figs. 3c,4c	2 → 3	3510.6	$^5I_6$ (2) Γ_1 (8673.4) → $^5I_7$ (3) Γ_1 (5162.8)
Fig. 4a	2 → 2	3517.65	$^5I_6$ (2) Γ_1 (8673.4) → $^5I_7$ (2) Γ_{34} (5155.75)
	3 → 3	3517.5	$^5I_6$ (3) Γ_{34} (8680.3) → $^5I_7$ (3) Γ_1 (5162.8)
Fig. 4b, 6b,c,d	1 → 2	3515.15	$^5I_6$ (1) Γ_2 (8670.9) → $^5I_7$ (2) Γ_{34} (5155.75)
Fig. 5	1 → 1	6089.3	$^5I_5$ (1) Γ_{34} (11241.6) → $^5I_7$ (1) Γ_2 (5152.3)
	1 → 2	6085.85	$^5I_5$ (1) Γ_{34} (11241.6) → $^5I_7$ (2) Γ_{34} (5155.75)
Fig. 7	2 → 1	6824.5	$^5F_5$ (2) Γ_{34} (15495.4) → $^5I_6$ (1) Γ_2 (8670.9)
	3 → 5	6824.95	$^5F_5$ (3) Γ_1 (15512.7) → $^5I_6$ (5) Γ_2 (8687.75)

4. Convolution of the Lorentzian lineshape with the instrumental function of a Fourier spectrometer

The shape of a Loretzian line can be written as follows:

$$F(\sigma-\sigma_0)=\frac{1}{1+[2(\sigma-\sigma_0)/W]^2} \qquad \text{(S1)}$$

Here σ_0 is the wavenumber of the line maximum and W is the full widths at half maximum (FWHM). The instrumental function of a Fourier spectrometer reads:

$$f_L(y)=\frac{\text{siny}}{y} \quad , \qquad y=4\pi L\sigma \qquad \text{(S2)}$$

The distance between the first zeros of f_L equals $\delta\sigma$ = 1/2L, where L is the maximal displacement of a moving mirror in a Michelson interferometer of a Fourier spectrometer ($\delta\sigma$ = 0.001 см$^{-1}$ in our case). FWHM equals 0.6 $\delta\sigma$.

The measured lineshape is determined by the convolution of the Loretzian (S1) and the instrumental function (S2):

$$Z(x,L)=\int\frac{1}{1+[2y/W]^2}\bullet\frac{\sin 4\pi L(y-x)}{4\pi L(y-x)}dy\,, \quad x=\sigma-\sigma_0 \qquad \text{(S3)}$$

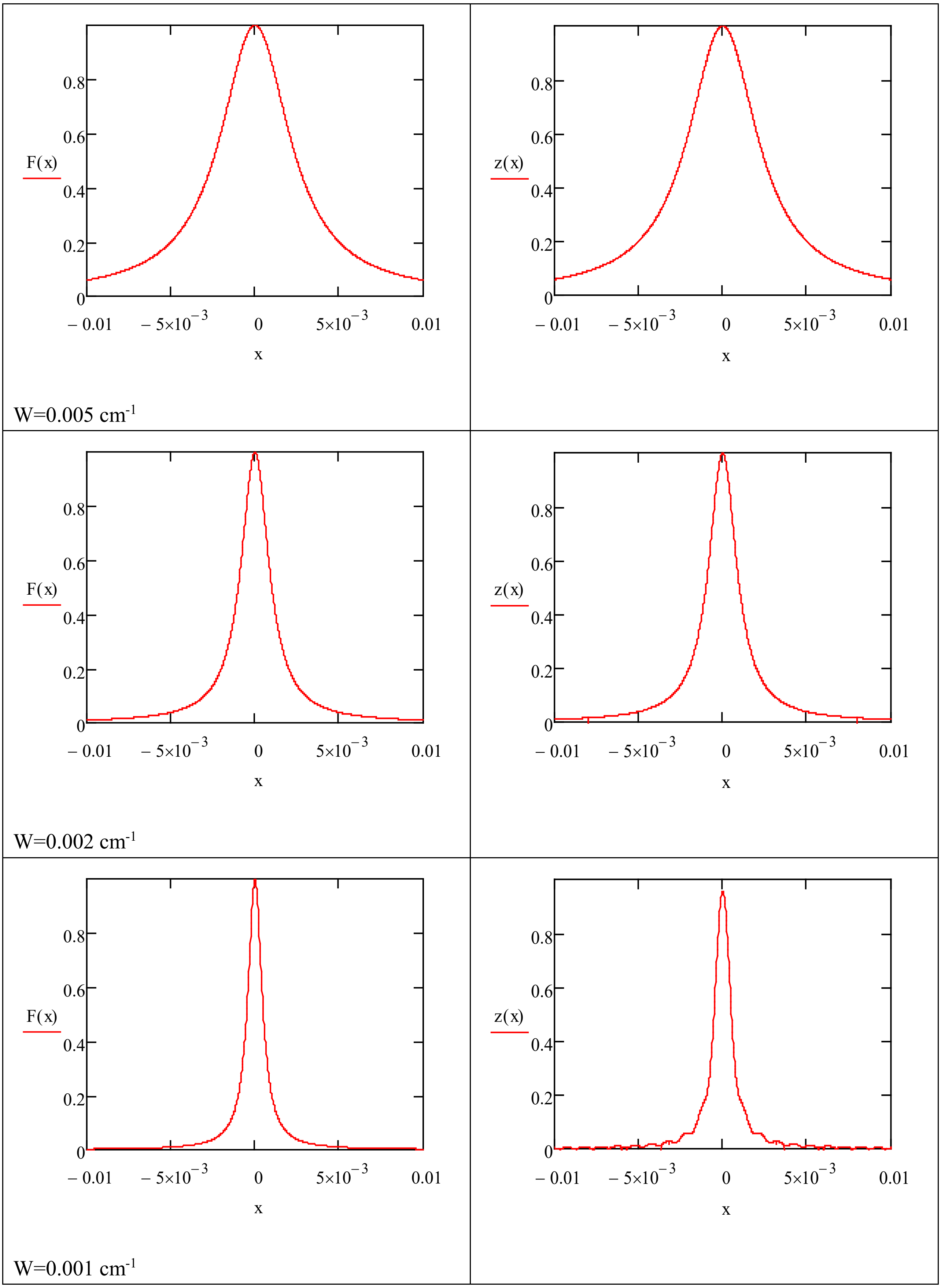

Figure S3. Lorentzian lines with different linewidths (FWHM) W (left column) and the measured line shapes using a Fourier spectrometer with the maximal displacement of a moving mirror L = 500 cm. The distance between the first zeros of the non-apodized instrumental function is $\delta\sigma = 1/2L = 0.001$ cm^{-1}

5. Magnetic *g* factors and their relation to intervals in the magnetic hyperfine structure

Magnetic g factor of a doublet level is defined by the relation

$$\Delta = \mu_B\, g\, B \tag{S4}$$

where Δ is a splitting of the doublet in a magnetic field B and $\mu_B = 0.4669$ cm^{-1}T^{-1} is the Bohr magneton.

The Γ_{34} doublets of Ho^{3+} in $LiYF_4$: Ho^{3+} split only in a magnetic field directed along the c (z) axis of the crystal, and the g factor $g_z = g_{||}$ is proportional to the matrix element of the z component of the total electronic moment $\boldsymbol{J}$, $<\Gamma_3 | J_z | \Gamma_3> = - <\Gamma_4 | J_z | \Gamma_4>$:

$$g_{||} = 2g_0 <\Gamma_3 | J_z | \Gamma_3> \tag{S5}$$

where g_0 is the Lande factor,

$$g_0 = 1+ \{J(J+1)-L(L+1)+S(S+1)\}\{2J(J+1)\}^{-1} \tag{S6}$$

The main contribution to the hyperfine structure (HFS) of the Γ_{34} crystal-field (CF) levels comes from the magnetic dipole hyperfine interaction

$$V = A_J\, \boldsymbol{J}\boldsymbol{I} \tag{S7}$$

where A_J is the magnetic hyperfine constant and $\boldsymbol{I}$ is the nuclear total moment, $I = 7/2$ for the nucleus of the only holmium isotope ^{165}Ho. The Hamiltonian (S7) results in eight equidistant electronic-nuclear components with energies

$$E = A_J <\Gamma_3 | J_z | \Gamma_3> m = A_J <\Gamma_4 | J_z | \Gamma_4> (- m) \tag{S8}$$

where $-7/2 \le m \le 7/2$, and corresponding intervals between them

$$\Delta_{HFS} = A_J \,|<\Gamma_3 | J_z | \Gamma_3>| \tag{S9}$$

Comparing Eqs. S5 and S9, we obtain the following relation:

$$| g_{||} | = 2g_0\Delta_{HFS} / A_J \tag{S10}$$

Experimental values of g factors were determined using Eq. S4. The interaction with neighboring levels influences the magnetic-field behavior of different hyperfine components in a different way. We determined the g factors of the high-energy and low-energy hyperfine components, g_{he} and g_{le}, respectively, and then calculated the average g factor $<g>$. The result was averaged over several transitions between a given Γ_{34} doublet and different singlets. In such way, absolute values of the g factors given in Table 1 were found.

Signs of the g factors were determined by inspection of the $\Gamma_{34} \rightarrow \Gamma_{34}$ transitions. According to the selection rules, only $\Delta m = 0$ transitions are allowed, herewith $\Gamma_3 \leftrightarrow \Gamma_4$ electric dipole (ED) and $\Gamma_3 \rightarrow \Gamma_3$ and $\Gamma_4 \rightarrow \Gamma_4$ magnetic dipole (MD) transitions can be realized (see Table S1). Thus the hyperfine intervals of the $\Gamma_{34} \rightarrow \Gamma_{34}$ ED lines equal to the sum (difference) of hyperfine intervals, if the involved levels have the same (opposite) signs of the matrix elements $<\Gamma_3 | J_z | \Gamma_3>$.

6. PL spectra and PL intensity maps for different observed transitions. Magnetic *g* factors.

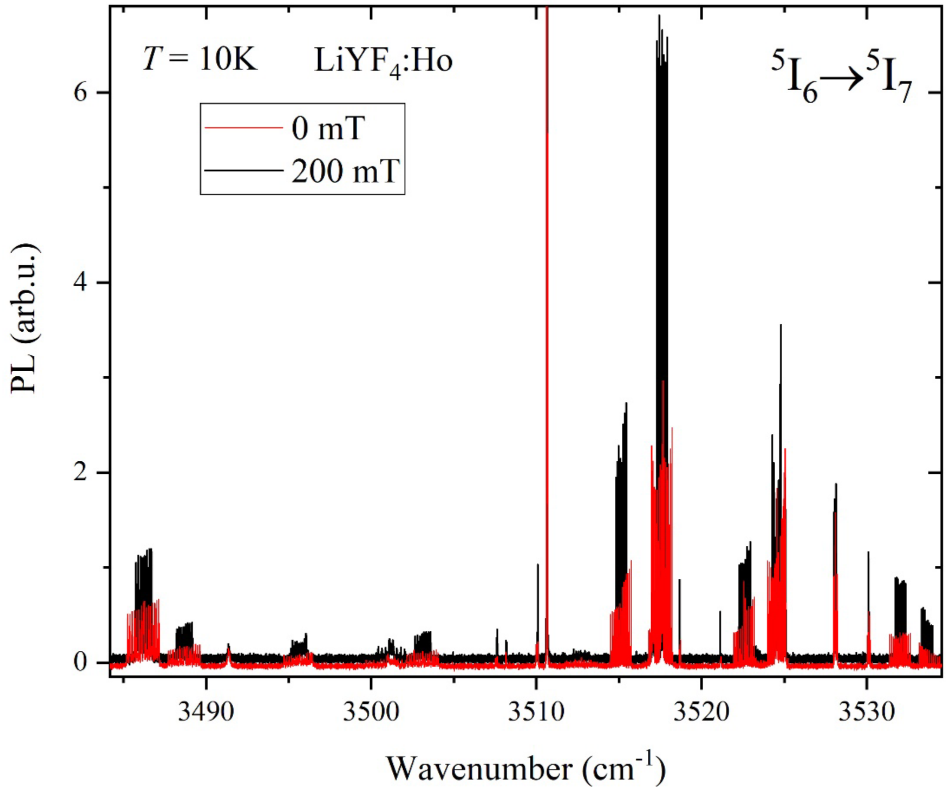

Figure S4. Photoluminescence spectra of $LiYF_4:Ho^{3+}$ (0.1 at. %) in the region of the $^5I_6 \rightarrow {}^5I_7$ optical transitions of Ho^{3+} at the temperature 10 K in a magnetic field $\boldsymbol{B}||c$: $B = 0$ (black) and $B = 200$ mT (red). $\lambda_{ex} = 638.3$ nm

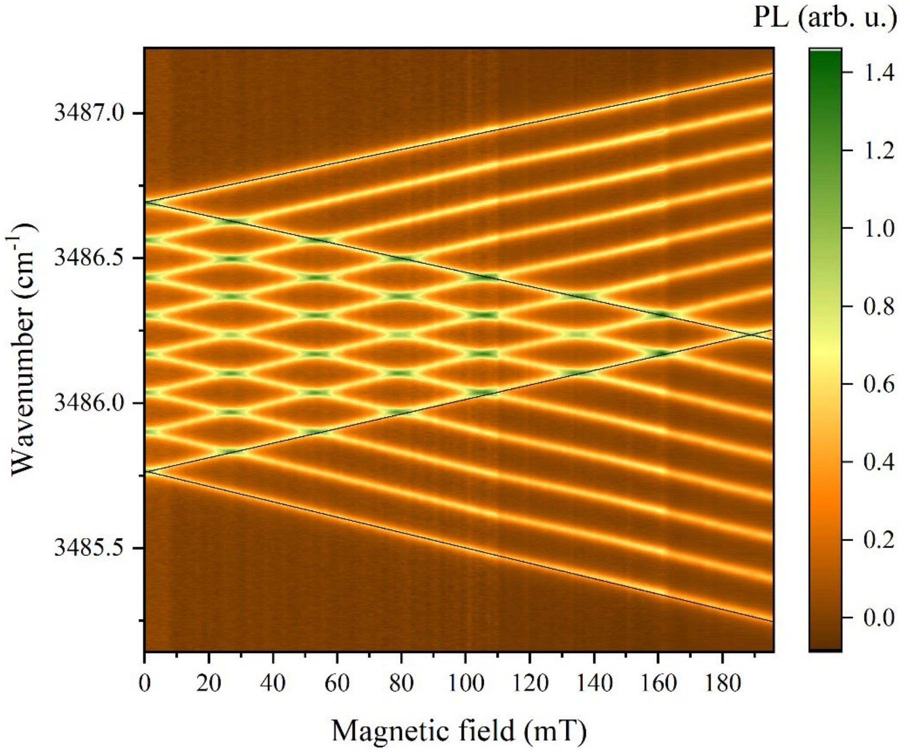

Figure S5. PL line 3486.2 cm^{-1} [$^5I_6\,\Gamma_2$ (8670.9) → $^5I_7\,\Gamma_{34}$ (5184.7)]. Δ_{HFS}=0.133 cm^{-1}, g_{he}=10.05, g_{le}=10.93, $\langle g \rangle$=10.49

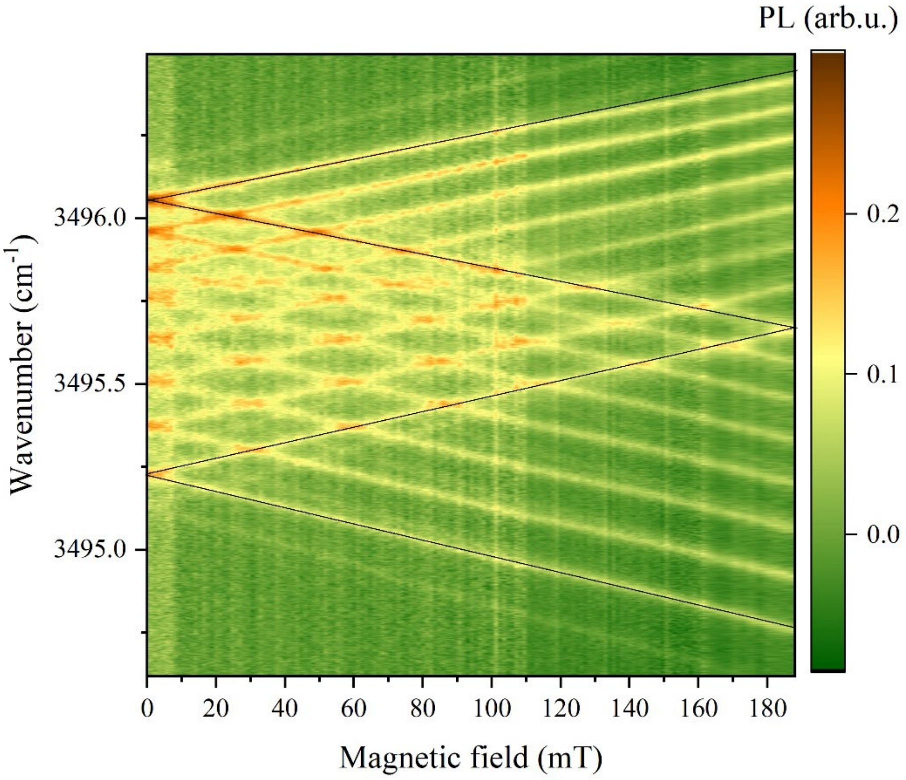

Figure S6. PL line 3495.6 cm^{-1} [$^5I_6\,\Gamma_{34}$ (8680.3) → $^5I_7\,\Gamma_{34}$ (5184.7)]. Δ_{HFS}=0.114 cm^{-1}, g_{he}=8.83, g_{le}=10.28, <g>=9.55. "Strong" "(weak") HFS corresponds to the difference (sum) of HFS intervals of the involved CF levels having opposite signs of the *g* factors and is formed by the ED (MD) transitions. Interaction of the CF level Γ_{34} (8680.3) with the neighboring Γ_{34} (8685.9) CF level results in squized hyperfine intervals in the high-frequency part of the spectrum.

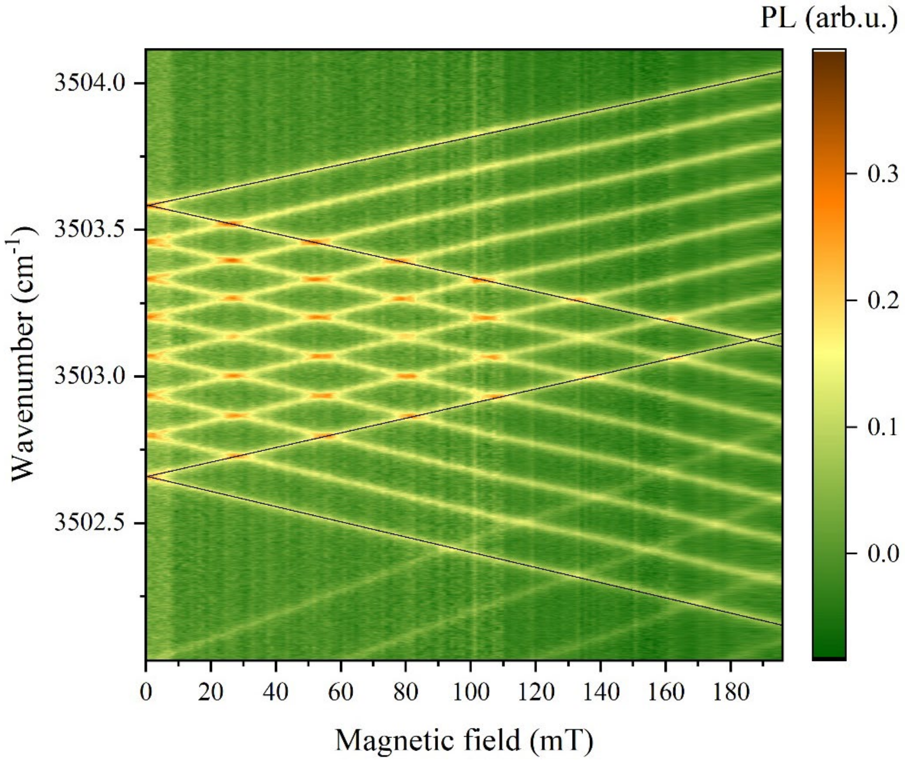

Figure S7. PL line 3503.05 cm^{-1} [$^5I_6\,\Gamma_2$ (8687.75) → $^5I_7\,\Gamma_{34}$ (5184.7)]. Δ_{HFS}=0.131 cm^{-1}, g_{he}=10.27, g_{le}=10.93, <g>=10.5

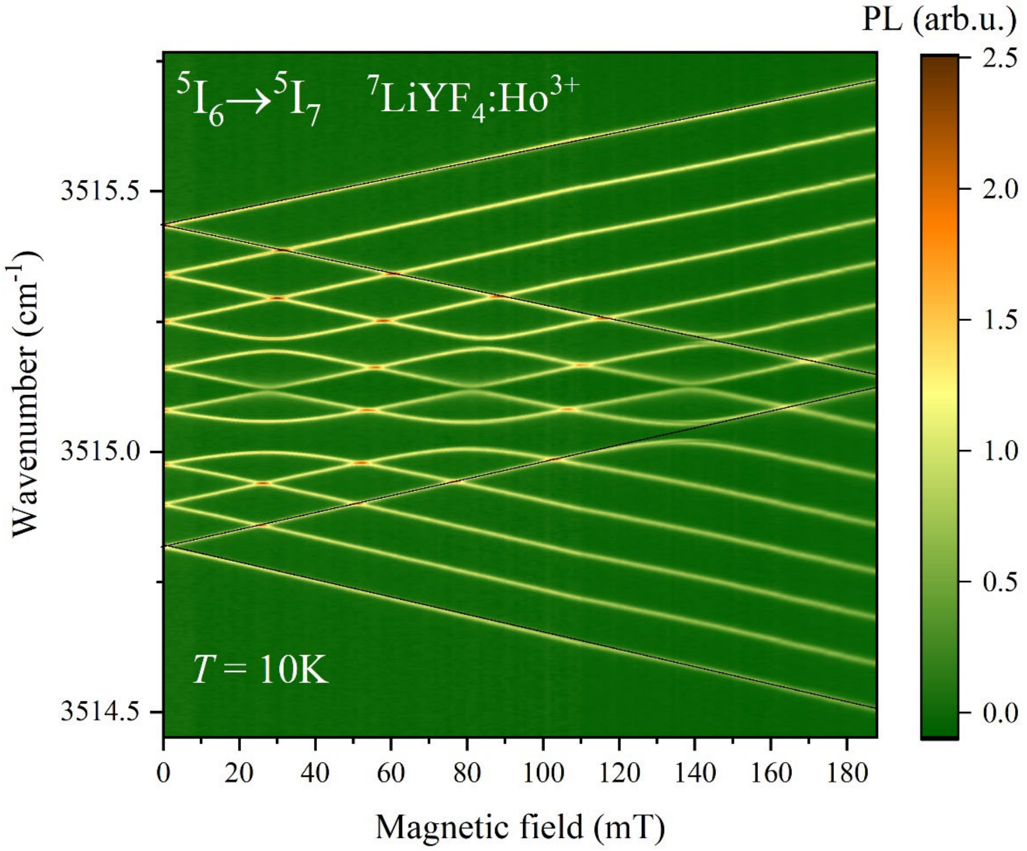

Figure S8. PL line 3515.15 cm^{-1} [$^5I_6\,\Gamma_2\,(8670.9) \rightarrow {}^5I_7\,\Gamma_{34}\,(5155.75)$]. Δ_{HFS}=0.088 cm^{-1}, g_{he}=6.46, g_{le}=7.097, $<g>$=6.778

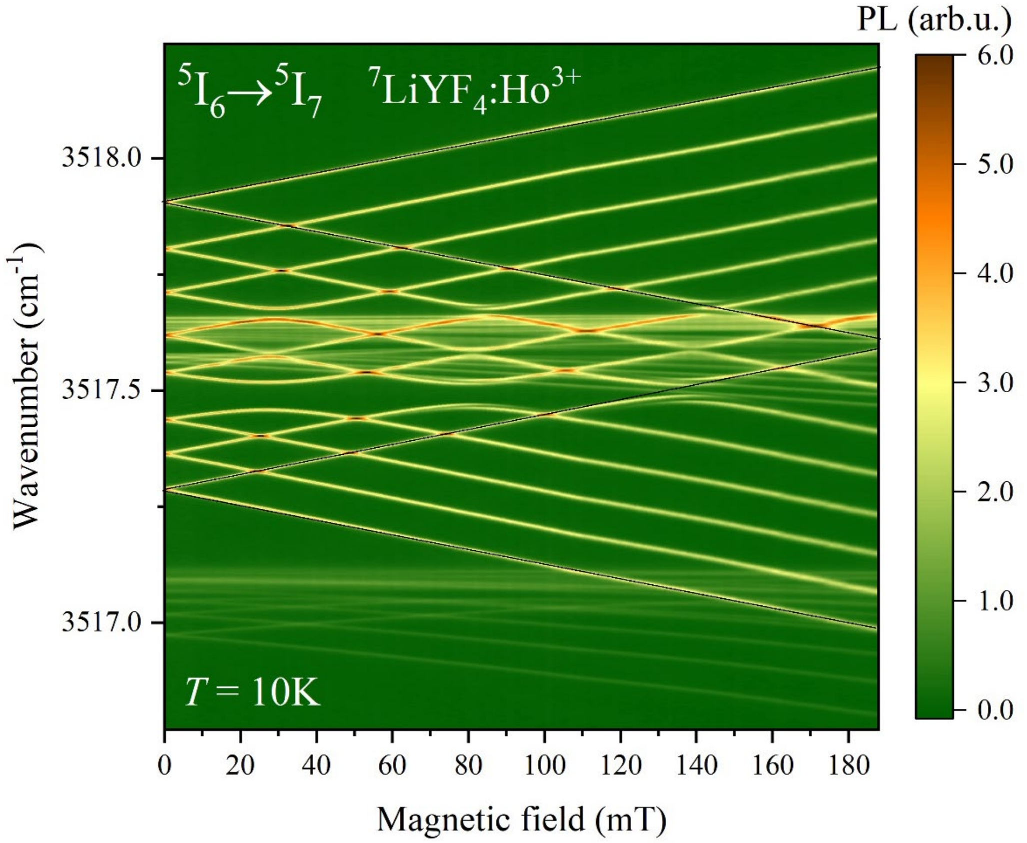

Figure S9. PL line 3517.6 cm^{-1}. [$^5I_6\,\Gamma_2\,(8673.3) \rightarrow {}^5I_7\,\Gamma_{34}\,(5155.75)$]. Δ_{HFS}=0.088 cm^{-1}, g_{he}=6.62, g_{le}=6.82, $<g>$=6.72

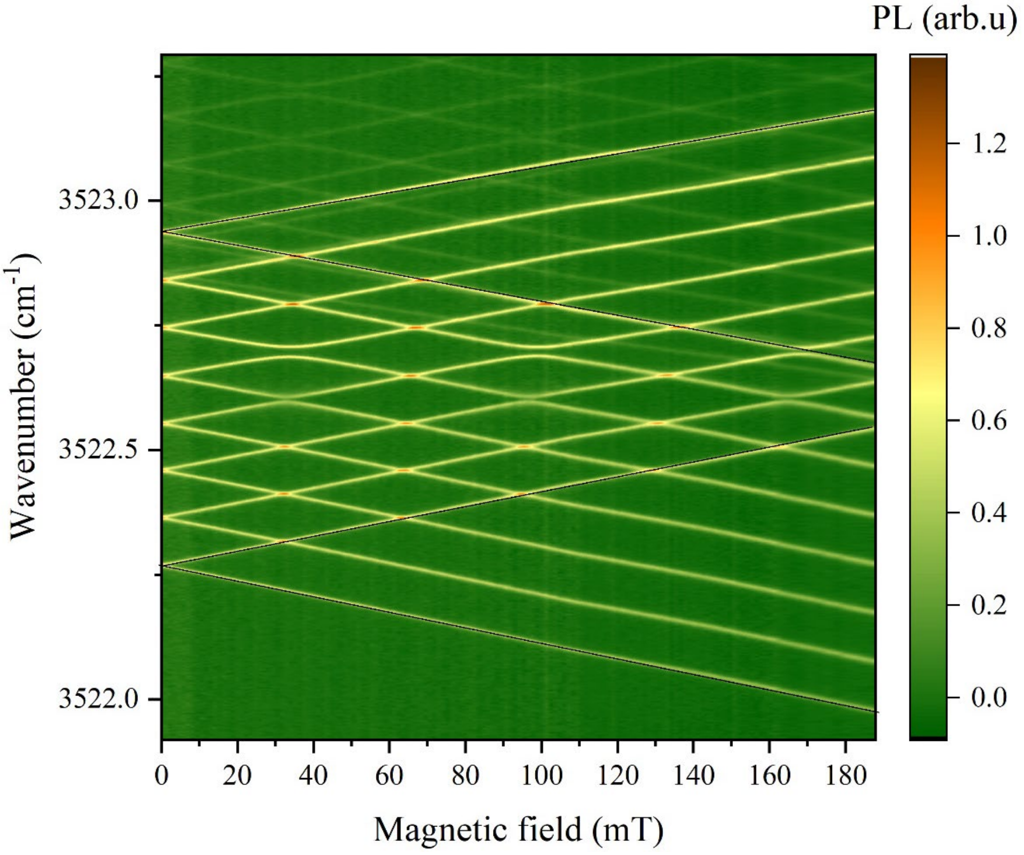

Figure S10. PL line 3522.6 cm^{-1}. [$^5I_6\,\Gamma_{34}$ (8685.9) → $^5I_7\,\Gamma_2$(5163.3)]. Δ_{HFS}=0.095 cm^{-1}, g_{he}=5.92, g_{le}=6.49, <g>=6.205

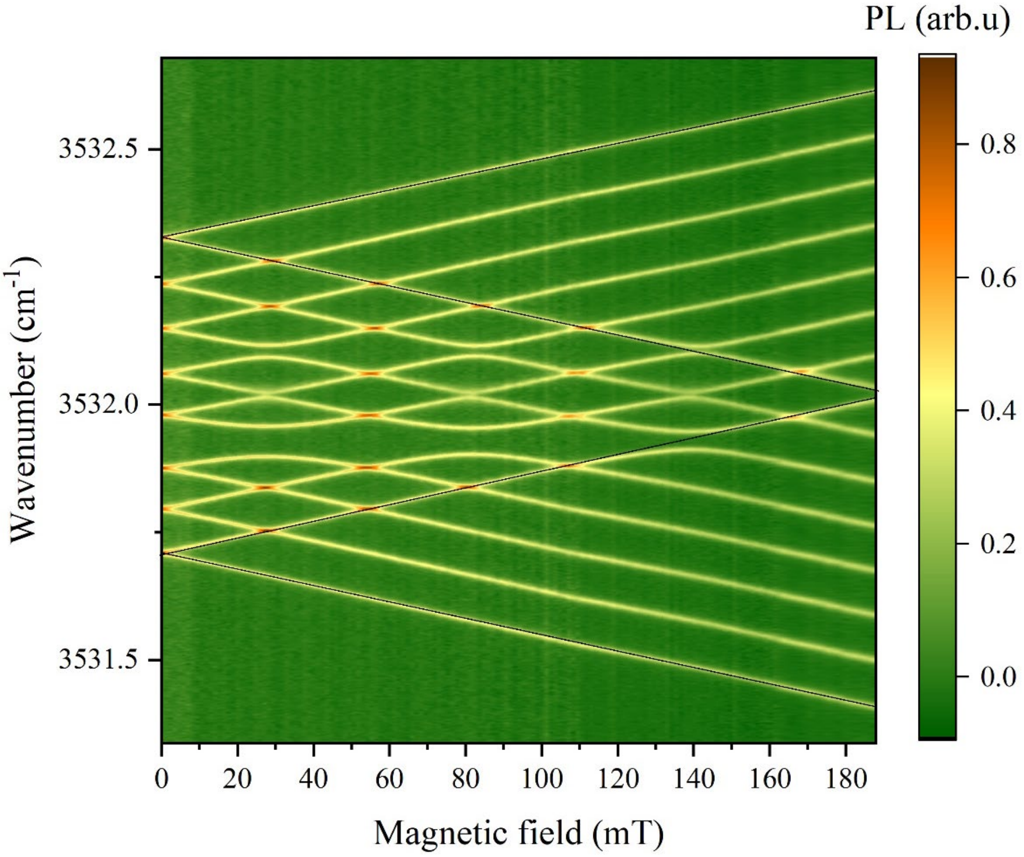

Figure S11. PL line 3532 cm^{-1}. [$^5I_6\,\Gamma_2$ (8687.75) → $^5I_7\,\Gamma_{34}$ (5155.75)]. Δ_{HFS}=0.088 cm^{-1}, g_{he}=6.62, g_{le}=6.87, <g>=6.745

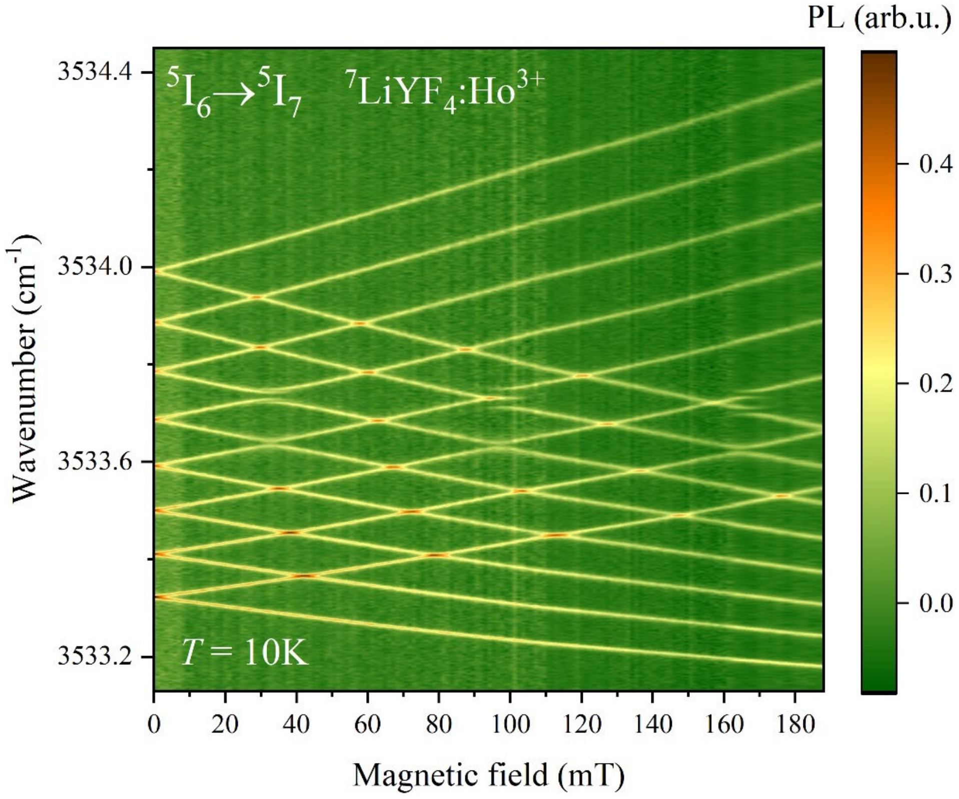

Figure S12. PL line 3533.6 cm^{-1}. [$^5I_6\,\Gamma_{34}\,(8685.9) \rightarrow {}^5I_7\,\Gamma_2\,(5152.3)$]. Δ_{HFS}=0.095 cm^{-1}, g_{he}=8.05, g_{le}=4.77, $\langle g \rangle$=6.41

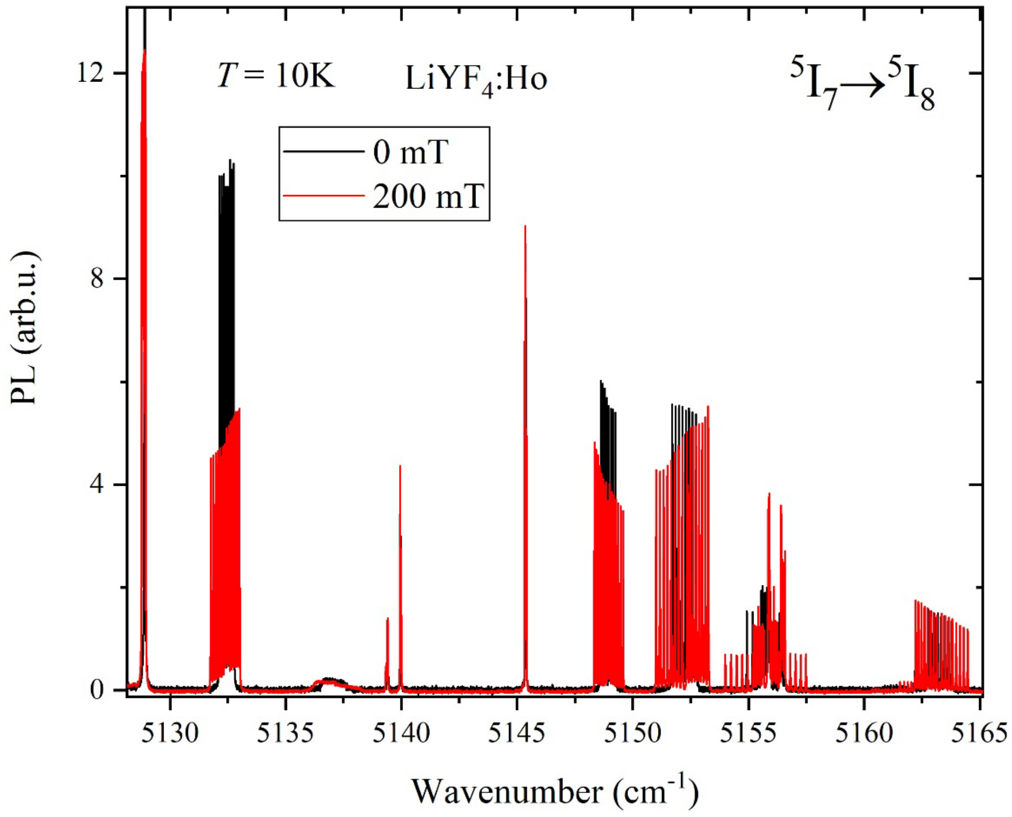

Figure S13. Photoluminescence spectra of $LiYF_4:Ho^{3+}$ (0.1 at. %) in the region of the $^5I_7 \rightarrow {}^5I_8$ optical transitions of Ho^{3+} at the temperature 10 K in a magnetic field $\boldsymbol{B}||c$: B = 0 (black) and B = 200 mT (red). λ_{ex} = 638.3 nm

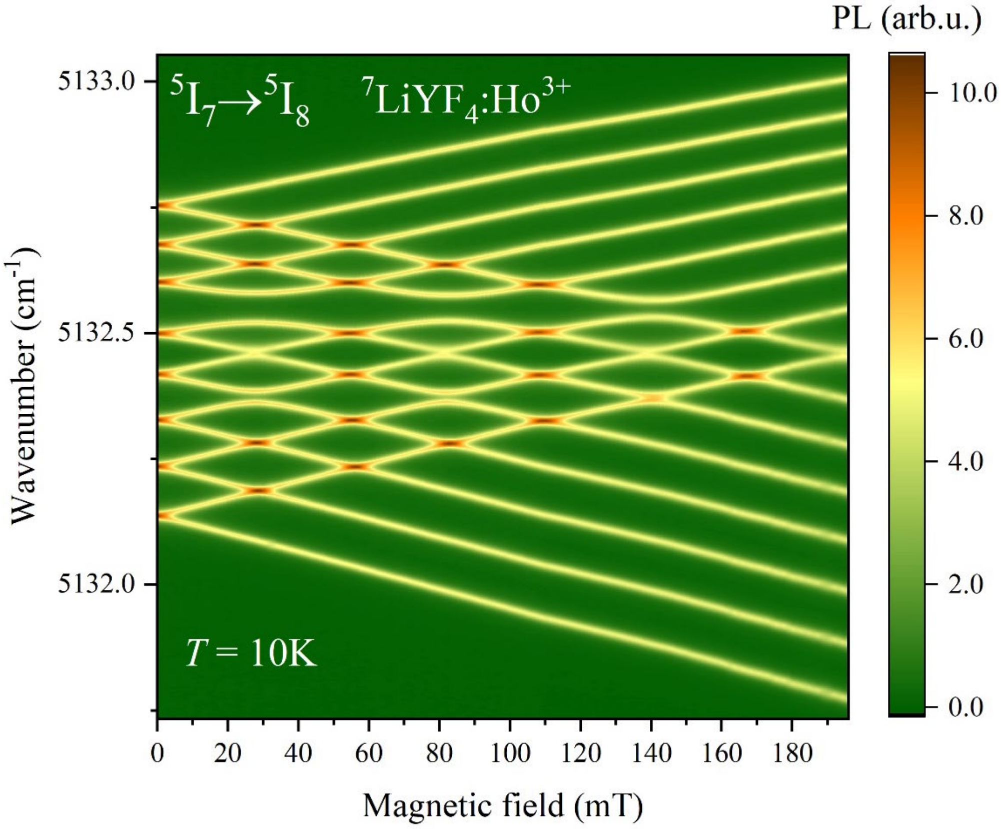

Figure S14. PL line 5132.45 cm^{-1}. [$^5I_7\,\Gamma_{34}$ (5155.75) → $^5I_8\,\Gamma_2$ (23.3)]. Δ_{HFS}=0.089 cm^{-1}, g_{he}=5.93, g_{le}=7.45, $\langle g \rangle$=6.69

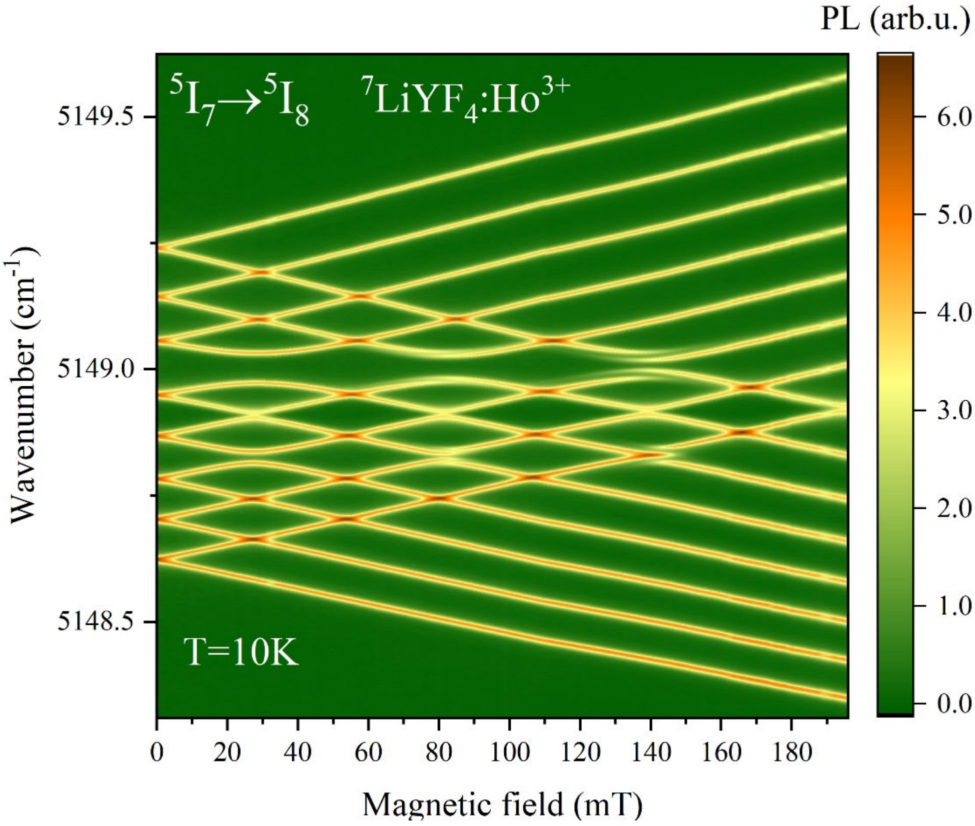

Figure S15. PL line 5148.9 cm^{-1}. [$^5I_7\,\Gamma_{34}$ (5155.75) → $^5I_8\,\Gamma_2$ (6.85)]. Δ_{HFS}=0.086 cm^{-1}, g_{he}=6.49, g_{le}=5.78, $\langle g \rangle$=6.135

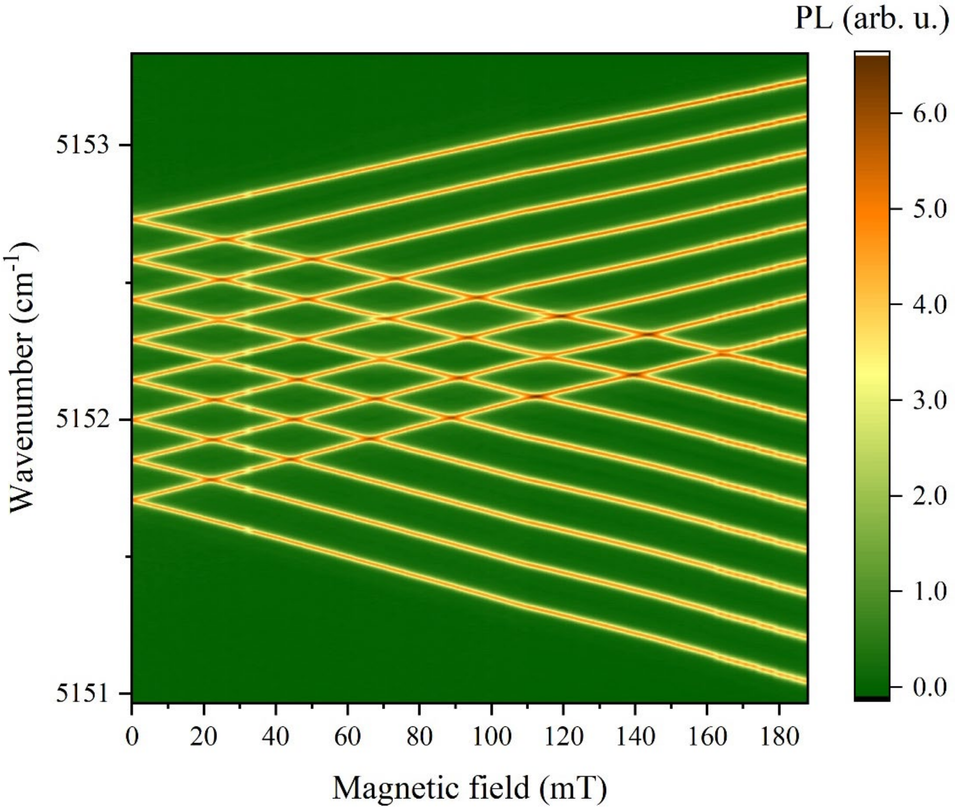

Figure S16. PL line 5152.3 cm^{-1}. [$^5I_7\,\Gamma_2$ (5152.3) → $^5I_8\,\Gamma_{34}$ (0)]. Δ_{HFS}=0.148 cm^{-1}, g_{he}=12.2 g_{le}=14.58, <*g*>=13.39. The observed HFS and magnetic *g* factor reflect the corresponding parameters of the ground state.

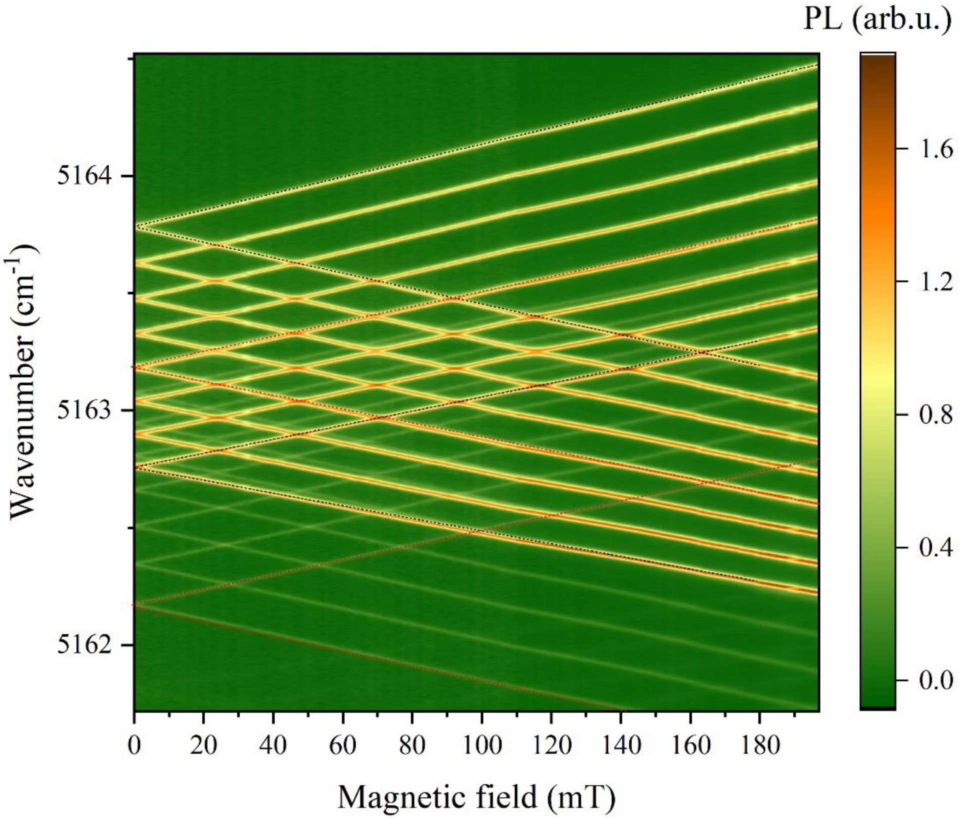

Figure S17. PL lines 5162.8 and 5163.3 cm^{-1}. [$^5I_7\,\Gamma_1$ (5162.8) → $^5I_8\,\Gamma_{34}$ (0) and [$^5I_7\,\Gamma_2$ (5163.3) → $^5I_8\,\Gamma_{34}$ (0)]. Δ_{HFS}=0.1457 cm^{-1}, g_{he}=14.48, g_{le}=12.39, <*g*>=13.435 and Δ_{HFS}=0.1444 cm^{-1}, g_{he}=13.34, g_{le}=13.65, <*g*>=13.495

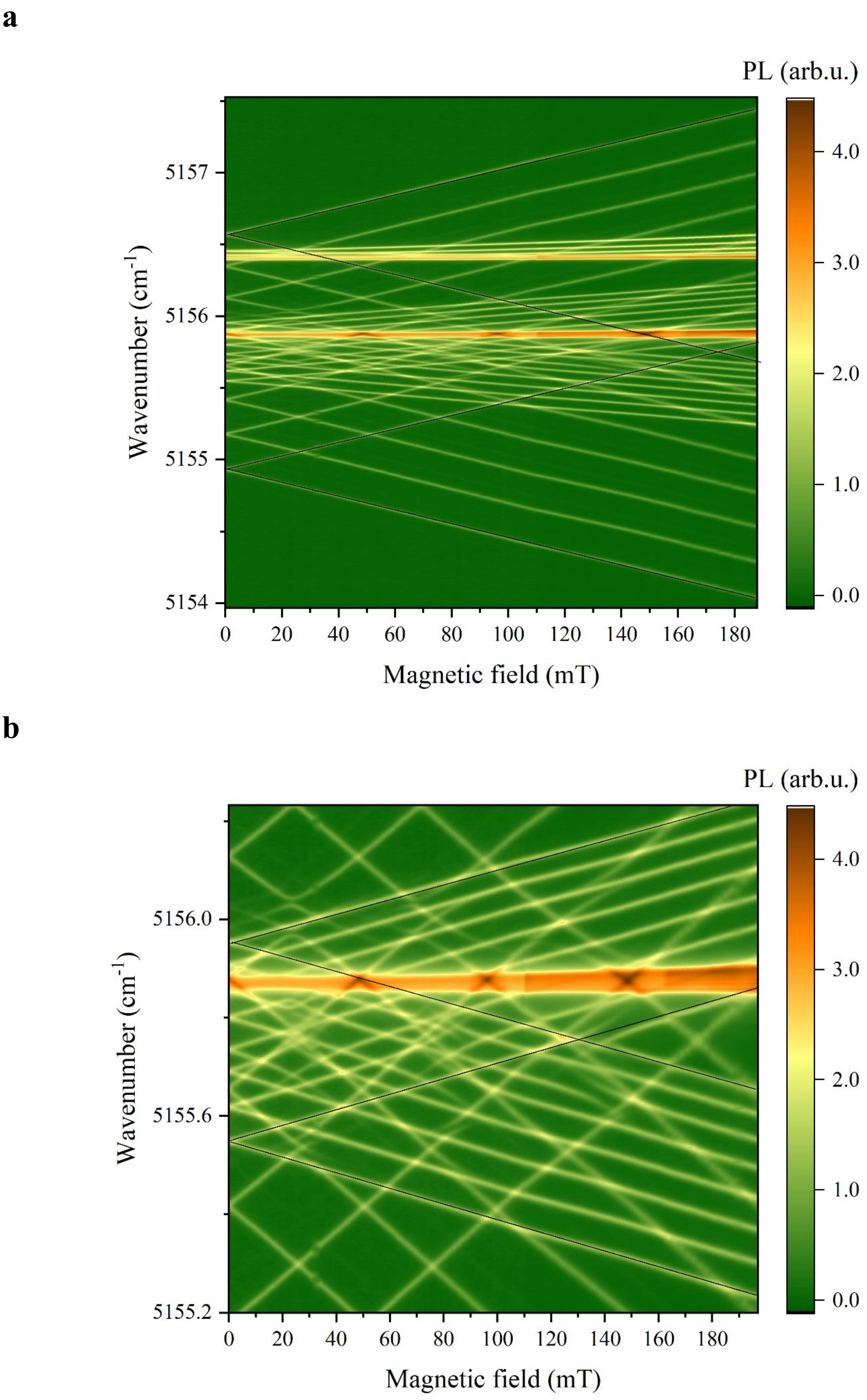

Figure S18. PL line 5155.75 cm^{-1}. [$^5I_7\,\Gamma_{34}\,(5155.75) \rightarrow\ ^5I_8\,\Gamma_{34}\,(0)$]. **a** Δ_{HFS}=0.238 cm^{-1}, g_{he}=19.94, g_{le}=20.28, $<g>$=20.11; **b** Δ_{HFS}=0.058 cm^{-1}, g_{he}=6.46, g_{le}=6.8, $<g>$=6.63

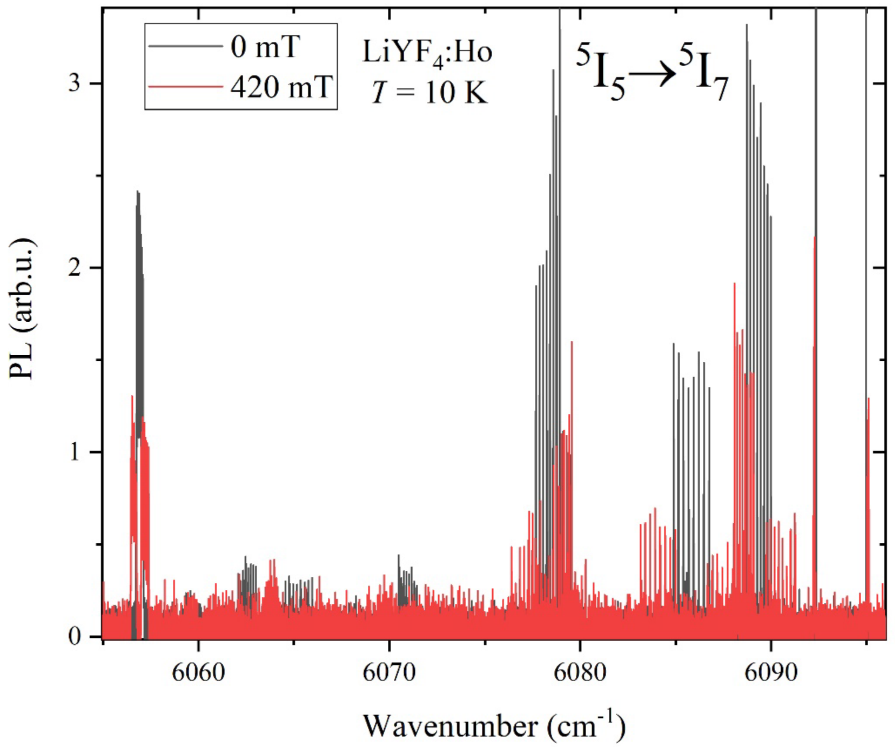

Figure S19. Photoluminescence spectra of $LiYF_4:Ho^{3+}$ (0.1 at. %) in the region of the $^5I_5 \rightarrow {}^5I_7$ optical transitions of Ho^{3+} at the temperature 10 K in a magnetic field $\boldsymbol{B}||c$: $B = 0$ (black) and $B = 420$ mT (red). $\lambda_{ex} = 638.3$ nm

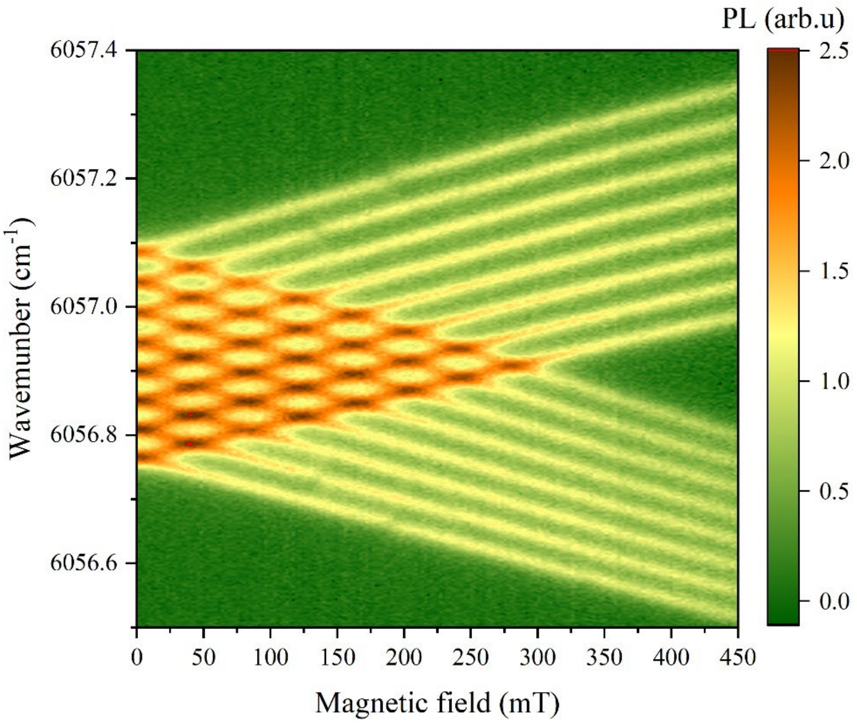

Figure S20. PL line 6056.9 cm^{-1}. [$^5I_5\,\Gamma_{34}\,(11241.6) \rightarrow {}^5I_7\,\Gamma_{34}\,(5184.7)$]. HFS corresponds to the difference of HFS intervals of the involved CF levels having opposite signs of the *g* factors and is formed by the ED transitions. Δ_{HFS}=0.047 cm^{-1}, g_{he}=2.7, g_{le}=2.315, $\langle g \rangle$=2.5. HFS corresponding to the sum of HFS intervals of the involved CF levels and allowed in the MD approximation is not observed, as $^5I_5 \rightarrow {}^5I_7$ MD transitions are forbidden in the free Ho^{3+} ions.

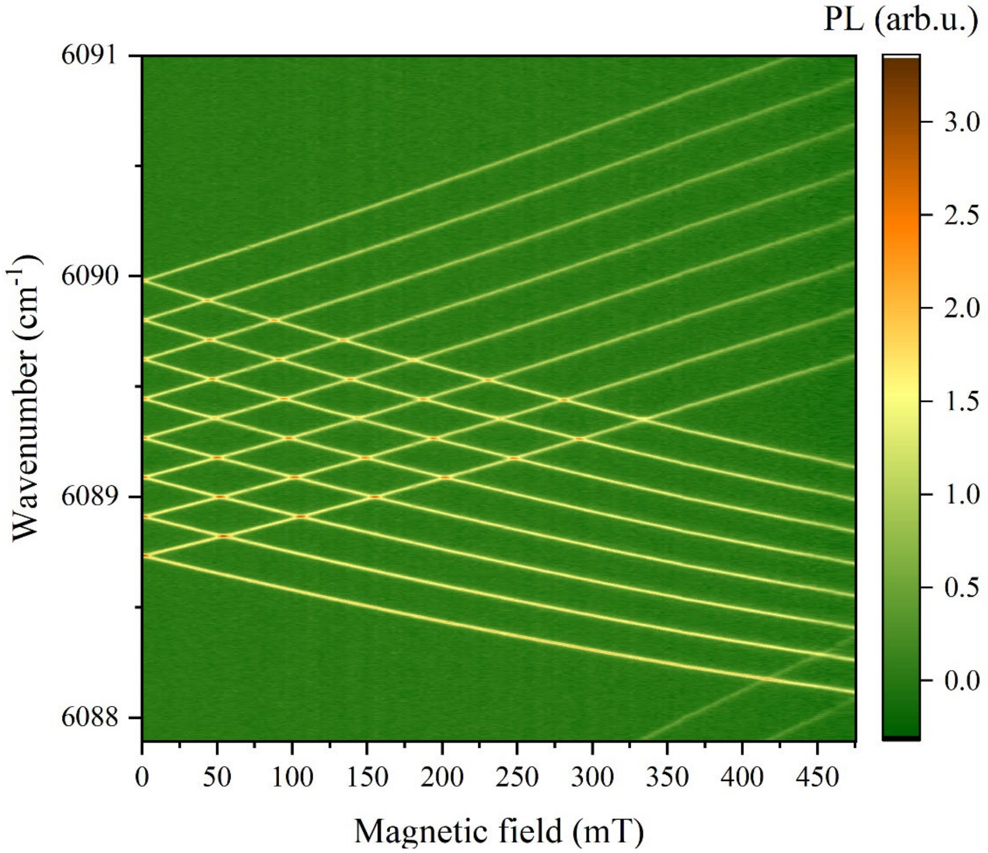

Figure S21. PL line 6089.3 cm^{-1}. [$^5I_5\,\Gamma_{34}$ (11241.6) → $^5I_7\,\Gamma_2$ (5152.3)]. Δ_{HFS}=0.179 cm^{-1}, g_{he}=8.9, g_{le}=7.08, $\langle g \rangle$=7.99

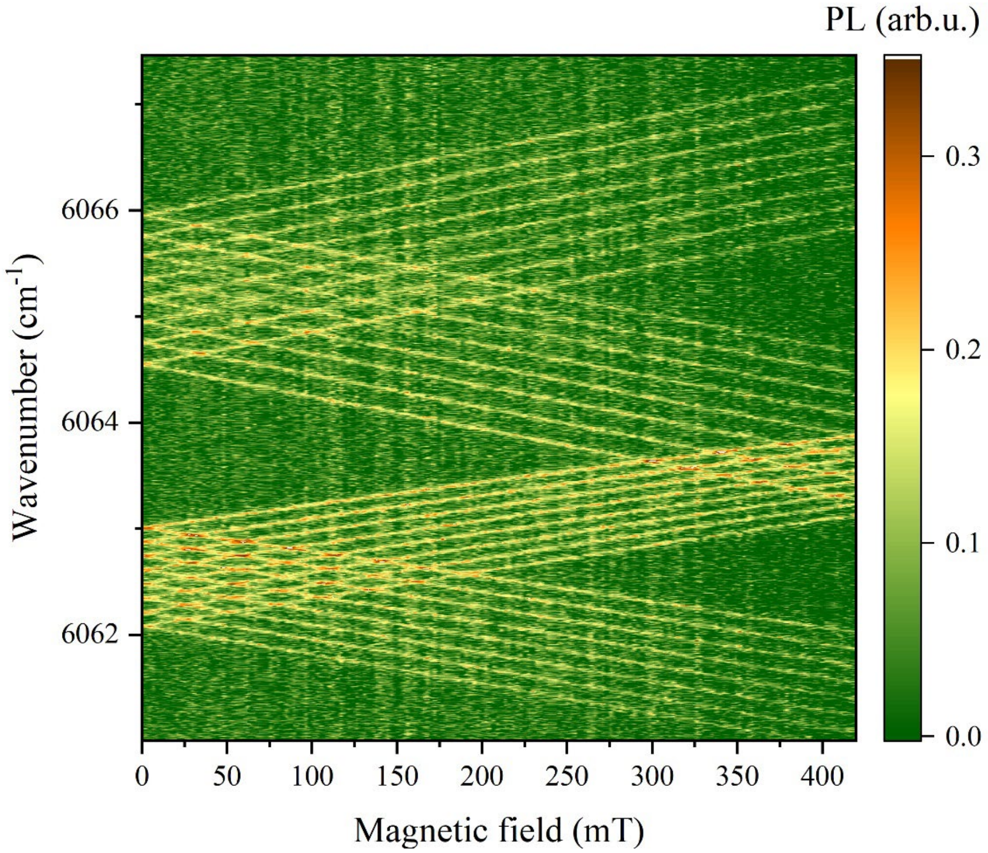

Figure S22. PL lines 6062.5 and 6065.2 cm^{-1}. [$^5I_5\,\Gamma_1$ (11247.2) → $^5I_7\,\Gamma_{34}$ (5184.7) and $^5I_5\,\Gamma_{34}$ (11249.9) → $^5I_7\,\Gamma_{34}$ (5184.7)]. Δ_{HFS}=0.27 cm^{-1}, g_{he}=14.77, g_{le}=14.67, $\langle g \rangle$=14.72 and Δ_{HFS}=0.207 cm^{-1}, g_{he}=13.27, g_{le}=12.84, $\langle g \rangle$=13.

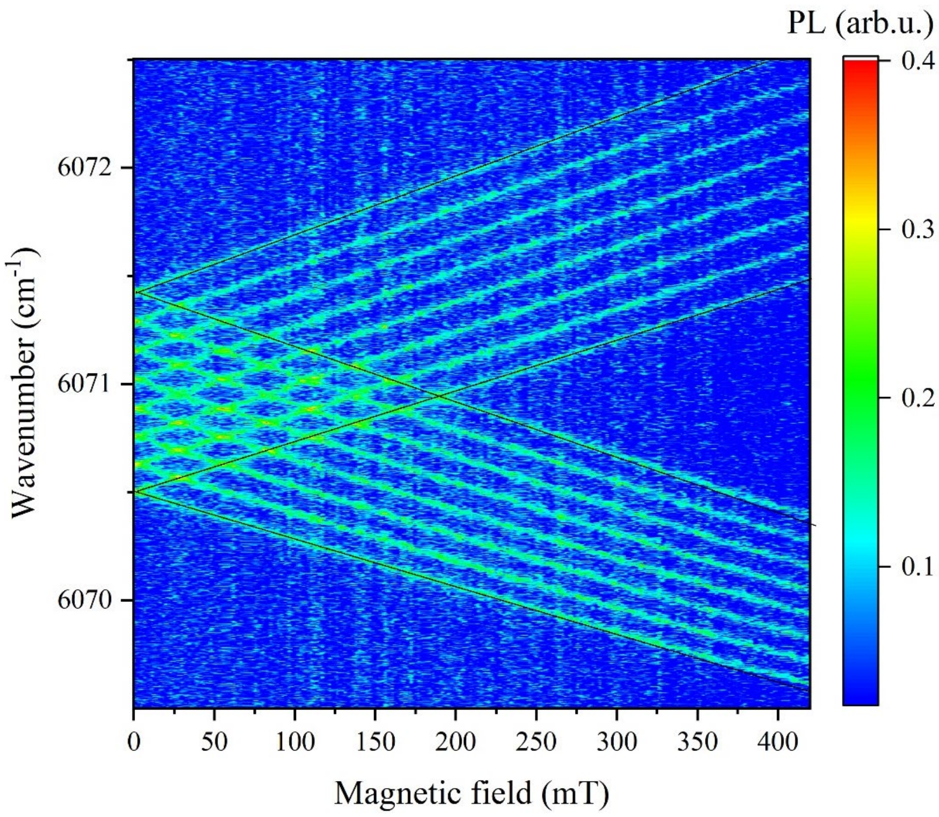

Figure S23. PL line 6070.9 cm^{-1}. [$^5I_5\,\Gamma_1$ (11255.6) → $^5I_7\,\Gamma_{34}$ (5184.7)]. Δ_{HFS}=0.136 cm^{-1}, g_{he}=11.2, g_{le}=9.49, $<g>$=10.345.

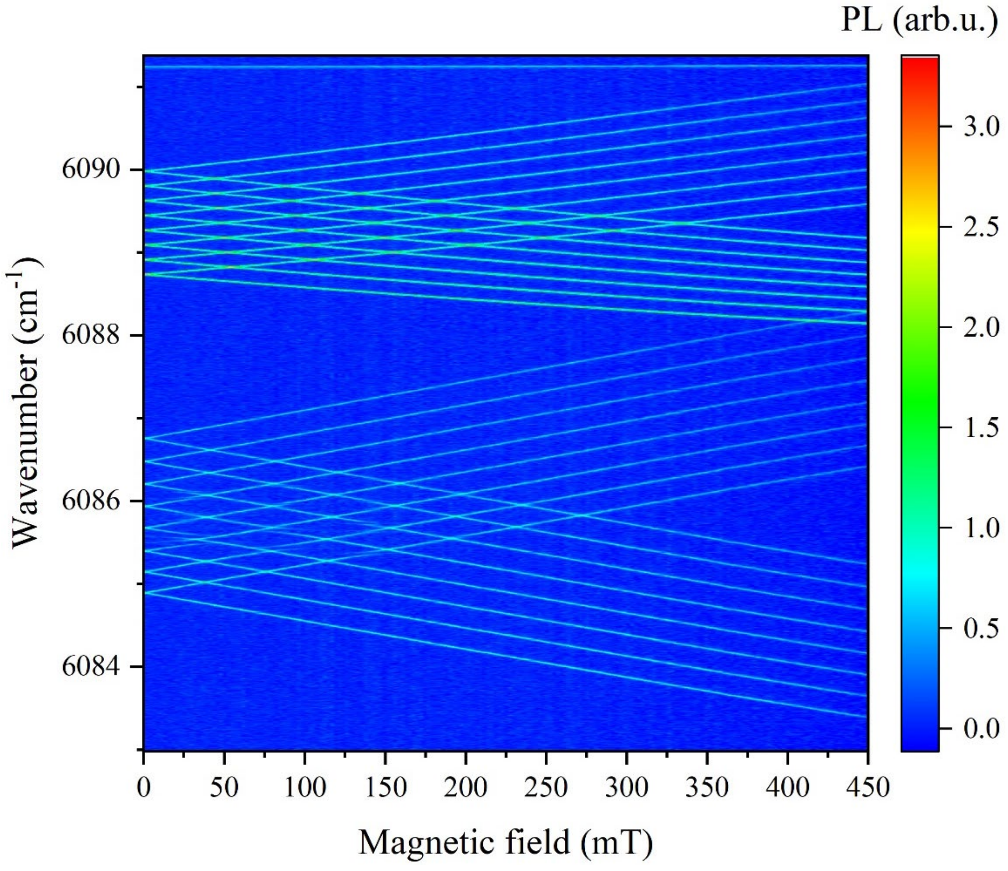

Figure S24. PL lines 6085.85 and 6089.3 cm^{-1}. [$^5I_5\,\Gamma_{34}$ (11241.6) → $^5I_7\,\Gamma_{34}$ (5155.75) and $^5I_5\,\Gamma_{34}$ (11241.6) → $^5I_7\,\Gamma_2$ (5152.3)]. Δ_{HFS}=0.27 cm^{-1}, g_{he}=14.77, g_{le}=14.67, $<g>$=14.72 and Δ_{HFS}=0.18 cm^{-1}, g_{he}=9.08, g_{le}=7.024, $<g>$=11.564

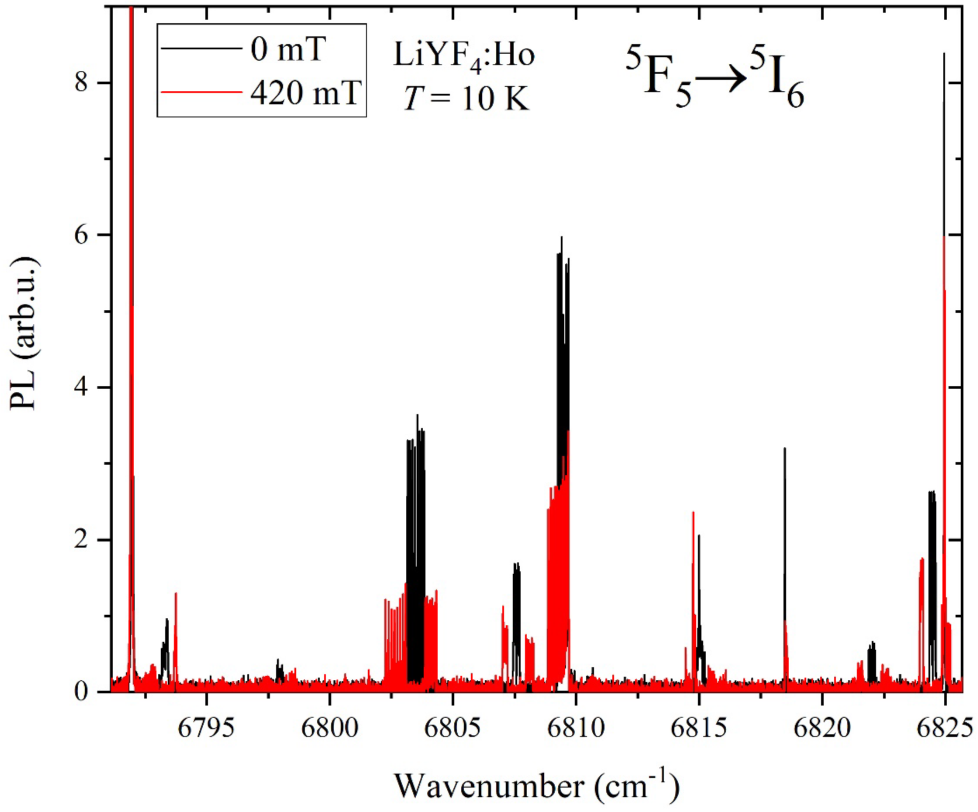

Figure S25. Photoluminescence spectra of $LiYF_4:Ho^{3+}$ (0.1 at. %) in the region of the $^5F_5 \rightarrow {}^5I_6$ optical transitions of Ho^{3+} at the temperature 10 K in a magnetic field $\boldsymbol{B}||c$: $B = 0$ (black) and $B = 420$ mT (red). $\lambda_{ex} = 638.3$ nm

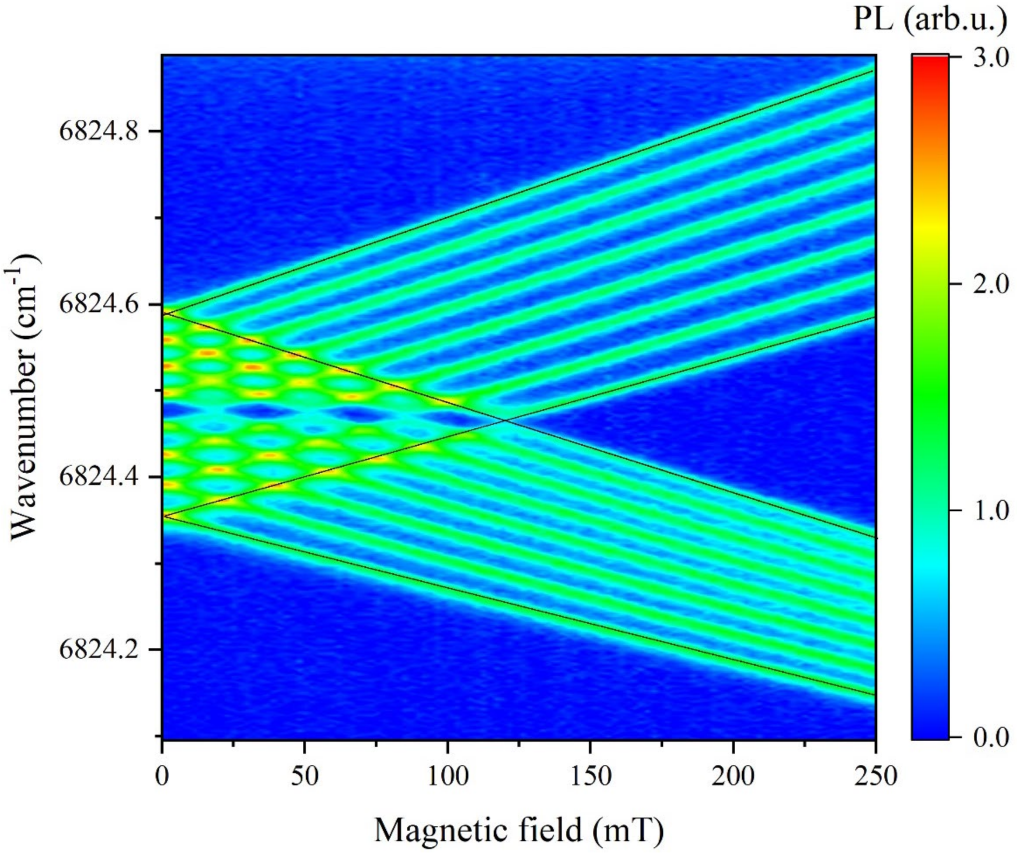

Figure S26. PL line 6824.5 cm^{-1}. [$^5F_5\,\Gamma_{34}$ (15495.4) $\rightarrow$ $^5I_6\,\Gamma_2$(8670.9)]. Δ_{HFS}=0.033 cm^{-1}, g_{he}=4.78, g_{le}=3.82, <g>=4.3

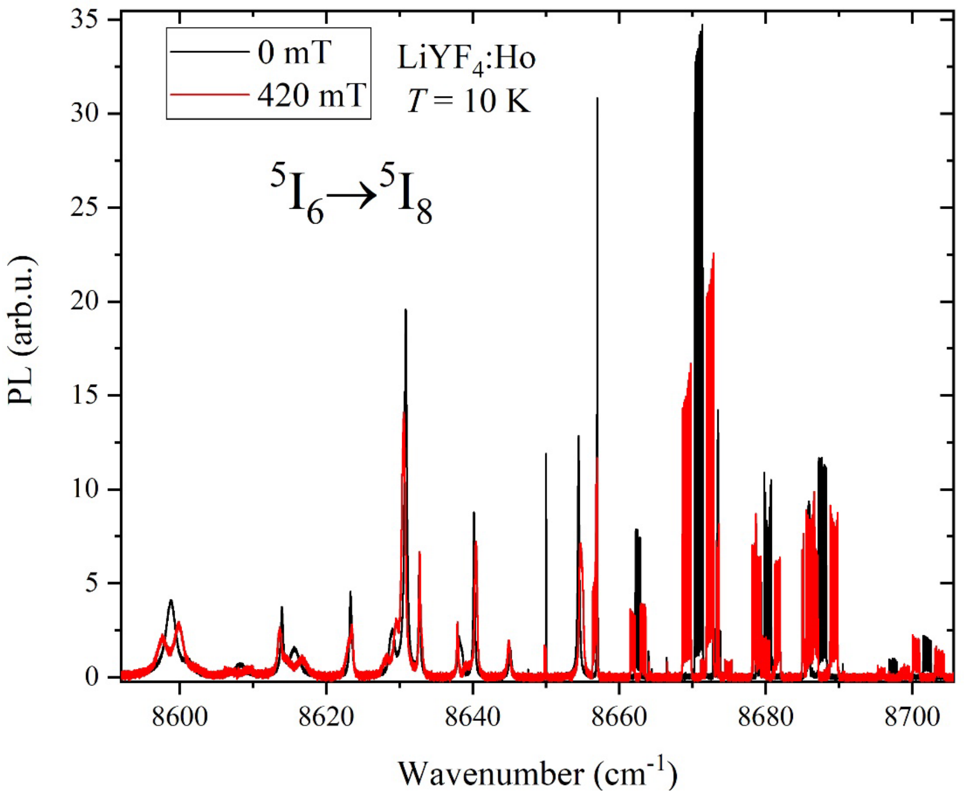

Figure S27. Photoluminescence spectra of $LiYF_4:Ho^{3+}$ (0.1 at. %) in the region of the $^5I_6 \rightarrow {}^5I_8$ optical transitions of Ho^{3+} at the temperature 10 K in a magnetic field $\boldsymbol{B}||c$: $B = 0$ (black) and $B = 420$ mT (red). $\lambda_{ex} = 638.3$ nm

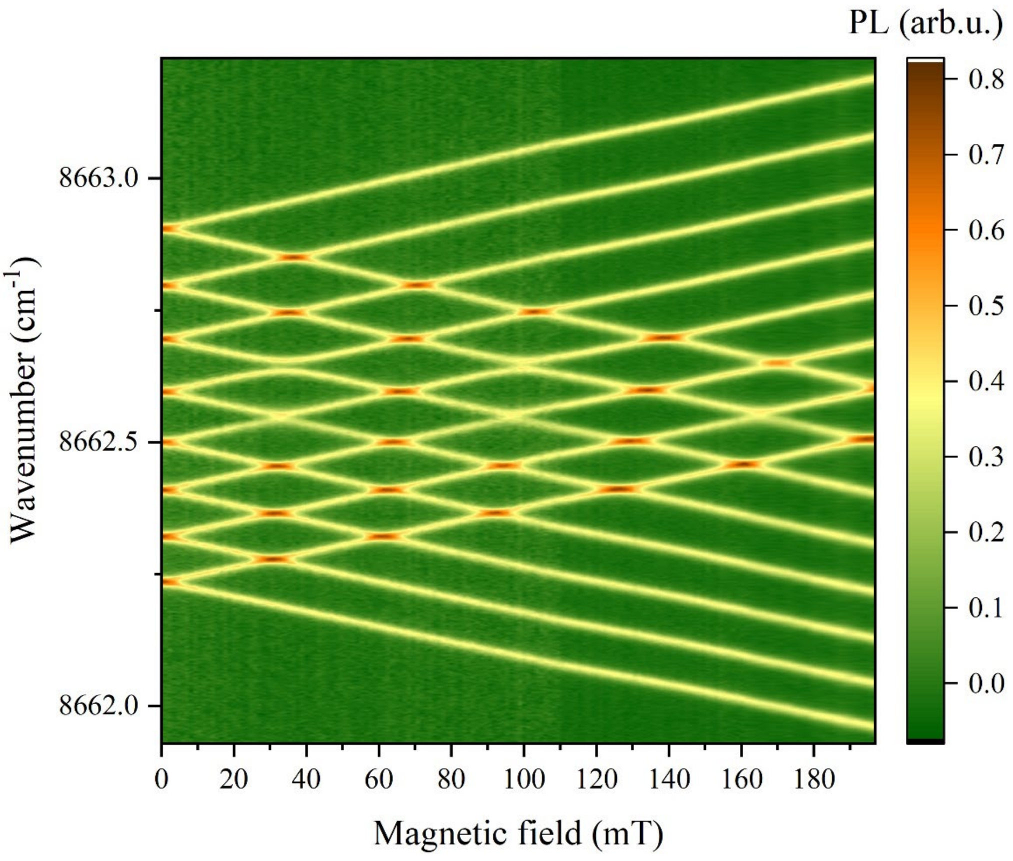

Figure S28. PL line 8762.6 cm^{-1}. [$^5I_6\,\Gamma_{34}$ (8685.9) ⟶ $^5I_8\,\Gamma_2$ (23.3)]. Δ_{HFS}=0.095 cm^{-1}, g_{he}=6.36, g_{le}=5.95, $<g>$=6.155

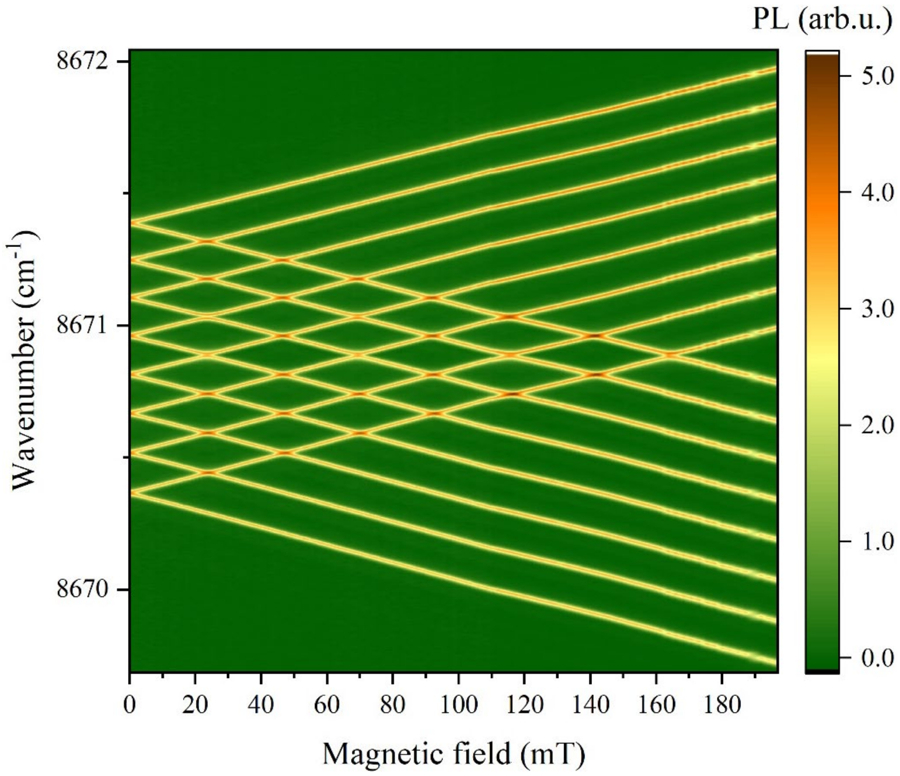

Figure S29. PL line 8670.9 cm^{-1}. [$^5I_6\,\Gamma_2$ (8670.9) $\rightarrow$ $^5I_8\,\Gamma_{34}$ (0)]. Δ_{HFS}=0.147 cm^{-1}, g_{he}=12.94, g_{le}=13.9, <g>=13.42. The observed HFS and magnetic g factor reflect the corresponding parameters of the ground state.

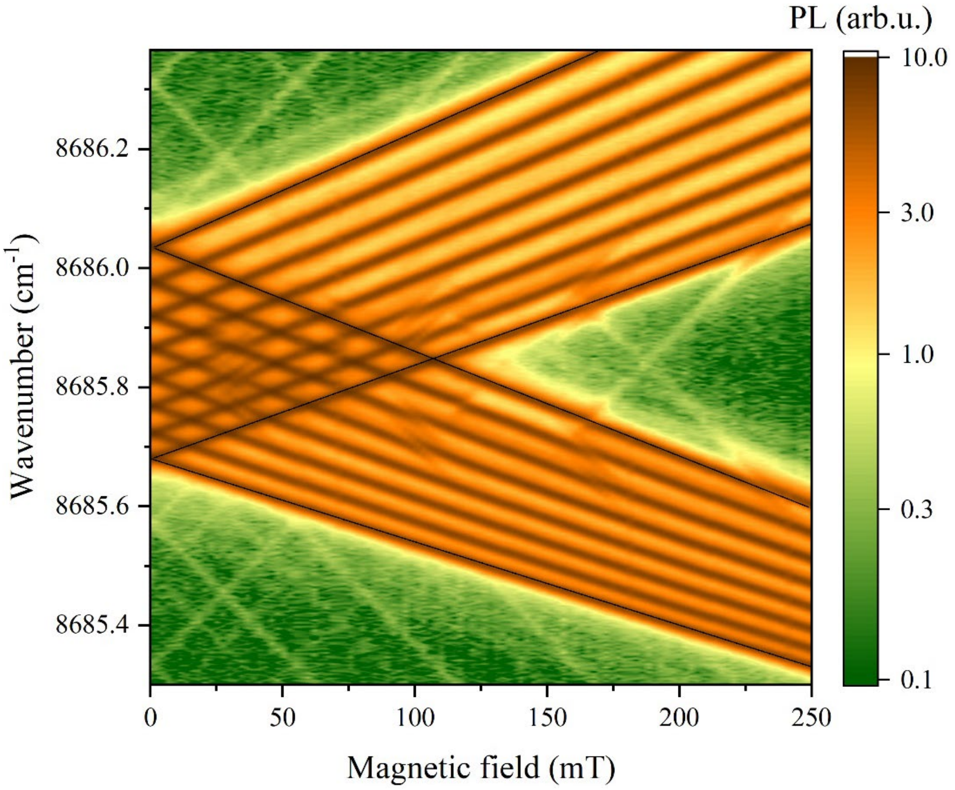

Figure S30. PL line 8685.9 cm^{-1}. [$^5I_6\,\Gamma_{34}$ (8685.9) $\rightarrow$ $^5I_8\,\Gamma_{34}$ (0)]. HFS corresponds to the difference of HFS intervals of the involved CF levels having opposite signs of the g factors and is formed by the ED transitions. Δ_{HFS}=0.052 cm^{-1}, g_{he}=7.3, g_{le}=5.87, <g>=6.585. HFS corresponding to the sum of HFS intervals of the involved CF levels and allowed in the MD approximation is very weak, as $^5I_6 \rightarrow {}^5I_8$ MD transitions are forbidden in the free Ho^{3+} ions.

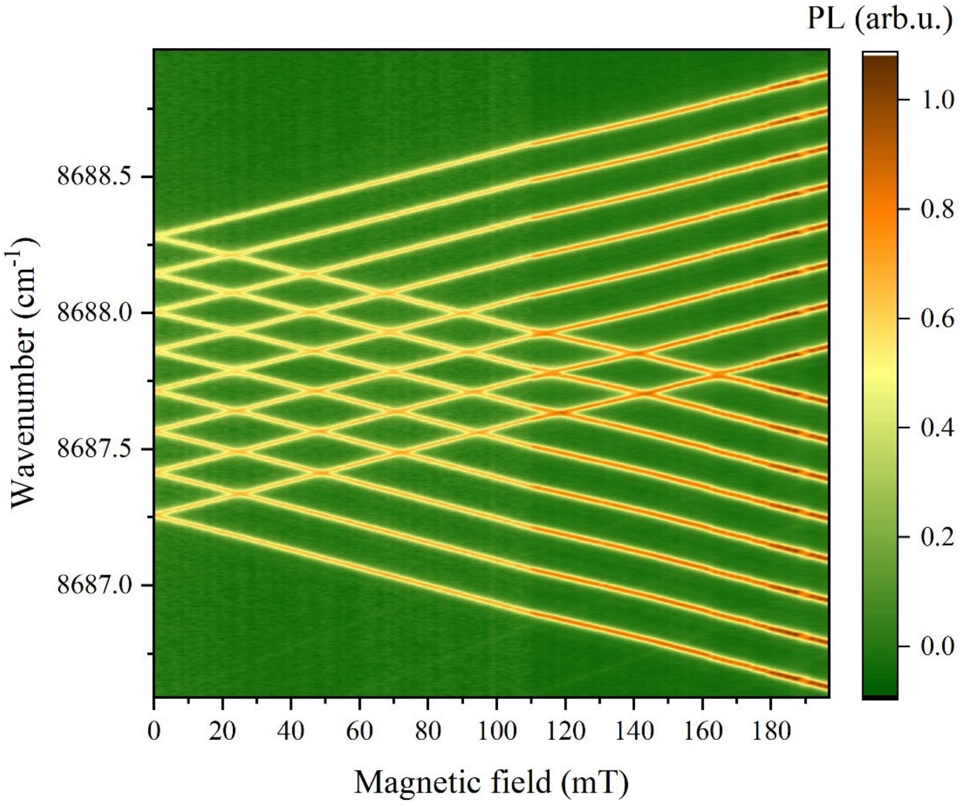

Figure S31. PL line 8787.75 cm^{-1}. [$^5I_6\,\Gamma_2$ (8787.75) → $^5I_8\,\Gamma_{34}$ (0)]. Δ_{HFS}=0.145 cm^{-1}, g_{he}=13.19, g_{le}=13.58, <g>=13.385. The observed HFS and magnetic g factor reflect the corresponding parameters of the ground state.

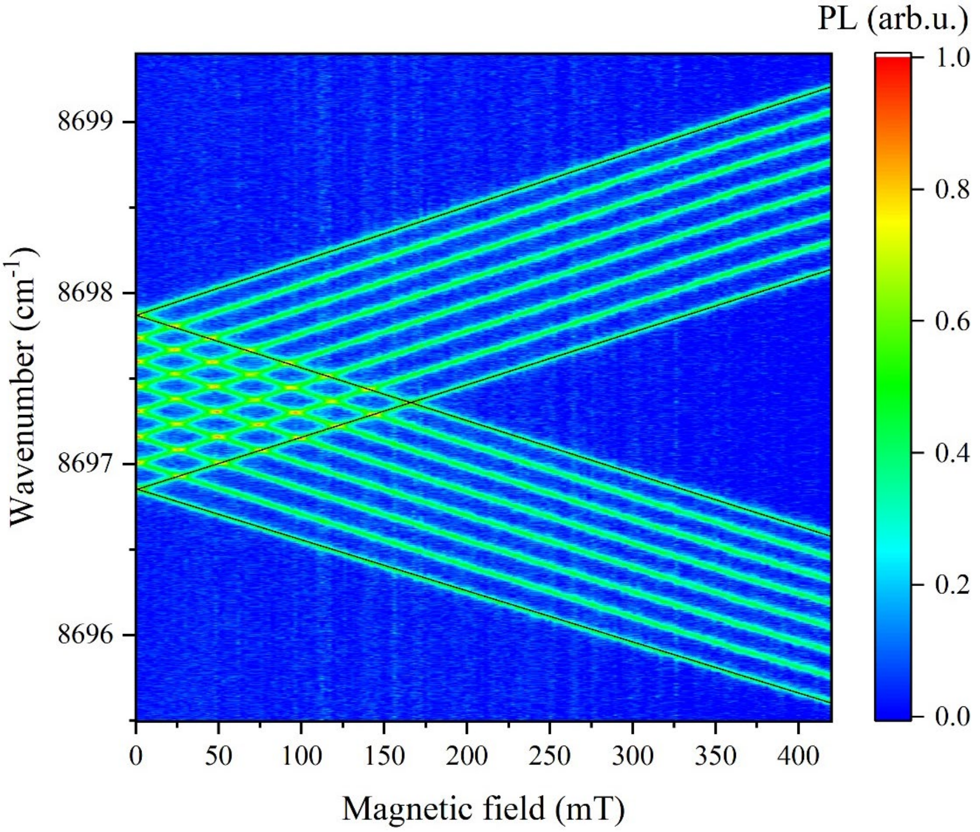

Figure S32. PL line 8797.4 cm^{-1}. [$^5I_6\,\Gamma_1$ (8797.4) → $^5I_8\,\Gamma_{34}$ (0)]. Δ_{HFS}=0.146 cm^{-1}, g_{he}=13.5, g_{le}=13, <g>=13.25. The observed HFS and magnetic g factor reflect the corresponding parameters of the ground state.

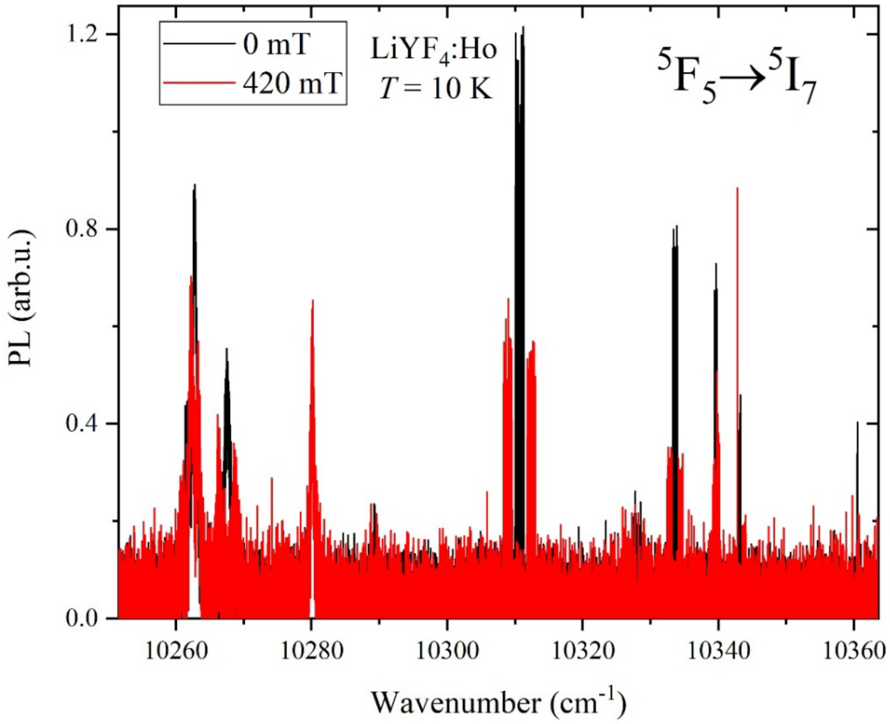

Figure S33. Photoluminescence spectra of $LiYF_4:Ho^{3+}$ (0.1 at. %) in the region of the $^5F_5 \rightarrow {}^5I_7$ optical transitions of Ho^{3+} at the temperature 10 K in a magnetic field $\boldsymbol{B}||c$: $B = 0$ (black) and $B = 420$ mT (red). $\lambda_{ex} = 638.3$ nm

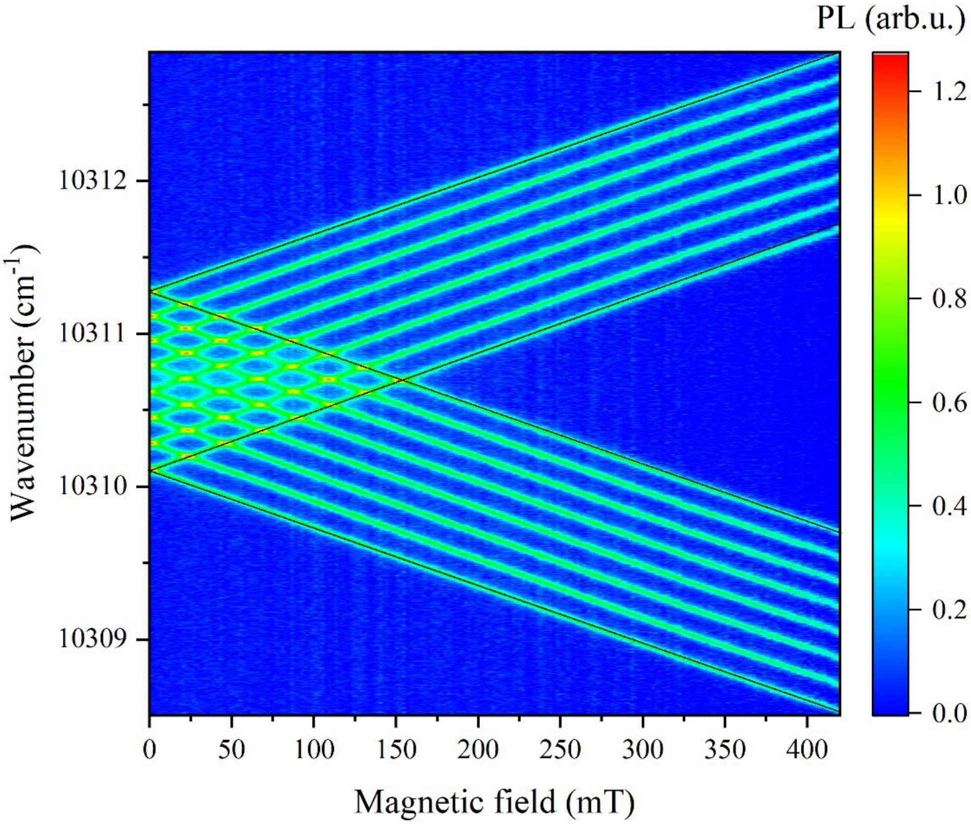

Figure S34. PL line 10310.7 cm^{-1}. [$^5F_5\,\Gamma_{34}$ (15495.4) $\rightarrow$ $^5I_7\,\Gamma_{34}$ (5184.7)]. HFS corresponds to the sum of HFS intervals of the involved CF levels having the same signs of the g factors and is formed by the ED transitions. Δ_{HFS}=0.17 cm^{-1}, g_{he}=14.46, g_{le}=14.67, $<g>$=14.56. HFS corresponding to the difference of HFS intervals of the involved CF levels and allowed in the MD approximation is not observed, as $^5F_5 \rightarrow {}^5I_7$ MD transitions are forbidden in the free Ho^{3+} ions.
